# Amplicon rearrangements during the extrachromosomal and intrachromosomal amplification process in a glioma

**DOI:** 10.1093/nar/gku1101

**Published:** 2014-11-06

**Authors:** Nicolas Vogt, Anne Gibaud, Frédéric Lemoine, Pierre de la Grange, Michelle Debatisse, Bernard Malfoy

**Affiliations:** 1 Institut Curie, Centre de Recherche, F-75248 Paris, France; 2 CNRS, UMR3244, F-75248 Paris, France; 3 UPMC, F-75248 Paris, France; 4 GenoSplice Technology, IUH, Hôpital Saint Louis, France

## Abstract

The mechanisms of gene amplification in tumour cells are poorly understood and the relationship between extrachromosomal DNA molecules, named double minutes (dmins), and intrachromosomal homogeneously staining regions (hsr) is not documented at nucleotide resolution. Using fluorescent *in situ* hybridization and whole genome sequencing, we studied a xenografted human oligodendroglioma where the co-amplification of the *EGFR* and *MYC* loci was present in the form of dmins at early passages and of an hsr at later passages. The amplified regions underwent multiple rearrangements and deletions during the formation of the dmins and their transformation into hsr. In both forms of amplification, non-homologous end-joining and microhomology-mediated end-joining rather than replication repair mechanisms prevailed in fusions. Small fragments, some of a few tens of base pairs, were associated in contigs. They came from clusters of breakpoints localized hundreds of kilobases apart in the amplified regions. The characteristics of some pairs of junctions suggest that at least some fragments were not fused randomly but could result from the concomitant repair of neighbouring breakpoints during the interaction of remote DNA sequences. This characterization at nucleotide resolution of the transition between extra- and intrachromosome amplifications highlights a hitherto uncharacterized organization of the amplified regions suggesting the involvement of new mechanisms in their formation.

## INTRODUCTION

In tumour cells, tens or hundreds of copies of a genome region may be observed. The mechanisms of formation of these amplifications are still poorly understood. Two forms of amplification are found: extrachromosomal DNA molecules, named double minutes (dmins), and intrachromosomal homogeneously staining regions (hsrs).

Dmins may comprise a single segment resulting from the circularization of a chromosome fragment, though, the fusion of several syntenic or non-syntenic DNA fragments is also observed (1–8). The transition from extrachromosomal to intrachromosomal amplification, recurrently observed in tumours and in experimental systems, indicates that one mechanism of formation of hsrs is the integration of dmins (9–13). Analysis of the junctions between the fragments suggests the involvement of non-homologous end-joining and microhomologous end-joining (NHEJ/MMEJ) mechanisms and chromosome fragmentation mechanisms are generally proposed to explain the formation of these complex structures (3,5–8,14–16), although a V(D)J-like illegitimate recombination has also been found (3). Replication-based mechanisms, such as Fork Stalling and Template Switching (FoSTeS) or microhomology-mediated break-induced replication (MMBIR), have also been proposed for germline and somatic complex rearrangements including amplifications (17–19).

Using fluorescent *in situ* hybridization (FISH) and whole genome sequencing approaches, we analysed at nucleotide resolution the organization of co-amplified sequences from the *EGFR* and *MYC* loci at successive passages of a xenografted human oligodendroglioma. Dmins were present at the early passages and an hsr was observed later. Our data support a mechanism of formation of the dmins and of their transformation into hsr driven by multiple rearrangements of the initial sequence resulting in the fusion of fragments of various lengths, some of them only a few tens of base pairs (bp) long. The sequence of the junctions showed that, in the dmins and in the hsr, NHEJ/MMEJ rather than replication repair mechanisms prevailed in their formation. Contigs formed of fragments from remote regions of the amplified regions were present. Clusters of breakpoints of a few hundred or thousand bp were observed but the fragments included in the contigs came from clusters hundreds of kilobases (kb) or megabases (Mb) apart. The characteristics of some pairs of junctions suggest that at least some of the fragments were not fused randomly but may have resulted from the concomitant repair of neighbouring breakpoints during the interaction of remote DNA sequences.

## MATERIALS AND METHODS

### Biological material

The oligodendroglioma ODA14 was from the Hôpital de la Salpêtrière (Paris). Informed written consent was obtained from the patient. Tumour was grown as a xenograft in athymic mice. Passages 1 and 2 grew slowly. Passage 3 grew with difficulty with a lot of cell death, and only a small living fragment was grafted in a single mouse where it finally grew correctly giving passage 4. The next passages grew correctly and the grafts were stopped at passage 20. Passages 2 and 4 were analysed. No material was available for cytogenetic or molecular analysis of passage 3.

### FISH

Cell preparations were obtained after short-term culture (1–2 days) of xenografted tumour fragments according to established procedures (20). Metaphase spreads were hybridized with BACs (BACPAC Resources, Oakland, CA, USA) or chromosome-specific paintings (Kreatech Diagnostics), as described previously (1) and in Supplementary Materials and Methods.

### Characterization of the amplified regions: whole genome sequencing

Amplifications of the *EGFR* and *MYC* genes in ODA14 were established by screening a series of glioma using quantitative polymerase chain reaction (PCR) (not shown). The Affymetrix Genome-Wide Human SNP Array 6.0 was first used to determine the extent of the amplifications. To delve deeper into the characterization of the amplified regions, whole genome sequencing was used. The libraries were prepared using Illumina TrueSeq sample preparation. The two samples were sequenced on an Illumina HiSeq 2000 in paired end mode with a read length of 100 bases. The sequencing resulted in about 100 million raw paired reads per sample corresponding to a coverage of ×10. Sequence data were recorded in the Sequence Read Archive database (Accession: ODA14p4, SRS476246; ODA14p2, SRS476247). For cluster generation we used the Illumina TruSeq PE Cluster Kit V3 and for sequencing the Illumina TruSeq SBS V3 Kit. The base calling, filtering of the data, index sorting and the adapter trimming were performed automatically by CASAVA Pipeline version 1.8.0. We first mapped the reads against human genome assembly hg19, using BWA v0.5.9-r16 (21). We then selected discordant reads using samtools v0.1.17 (22) and bedTools v2.14.2 (23). Discordant reads were aligned using novoalign v2.08.01 (Novoalign, www.novocraft.com), in two passes, once with the option ‘-r Random’ and once with the option ‘-r Ex’ in order to align missed concordant pairs in the first step. The remaining discordant mappings were processed with hydra-sv v0.5.3 (24). Finally, only breakpoints with more than two sequencing reads and falling into the amplified regions determined using Single-nucleotide polymorphism (SNP) data were selected. Junctions selected from the whole genome sequencing were confirmed by sequencing PCR products obtained using primers localized on both sides of the junctions. In some cases, sequences a few tens or hundreds of bp distant in the normal genome were involved in one arm of two different junctions. To look for the possible association of the two junctions in a contig, PCR between the two junctions were performed on the tumour DNA. When a fragment was amplified, it was sequenced to confirm the link. Step by step, this approach characterizes contigs comprising several segments. When no additional segments could be added to a contig using this approach, chromosome walking (Universal Genome Walker kit and Advantage GC Polymerase Mix, BD Biosciences) was used to search for other junctions using primers localized at the ends of the contig. The final structure was confirmed by PCR using primers designed in sequences localized at both ends of the contig. PCR products were sequenced by the conventional Sanger capillary sequencing method and compared with the reference genome to determine the exact position of the junctions.

Sequence data used in this work refer to the human genome sequence (hg19 released February 2009) available at the University of California Santa Cruz (UCSC) Genome Bioinformatics site (http://genome.ucsc.edu/) (25). Sequence comparisons were performed using BLAT software. Repeated sequences (low-copy repeats, segmental duplications and repetitive elements) were identified using the human genome sequence data.

## RESULTS

### FISH characterization of the amplifications

**Figure 1.**
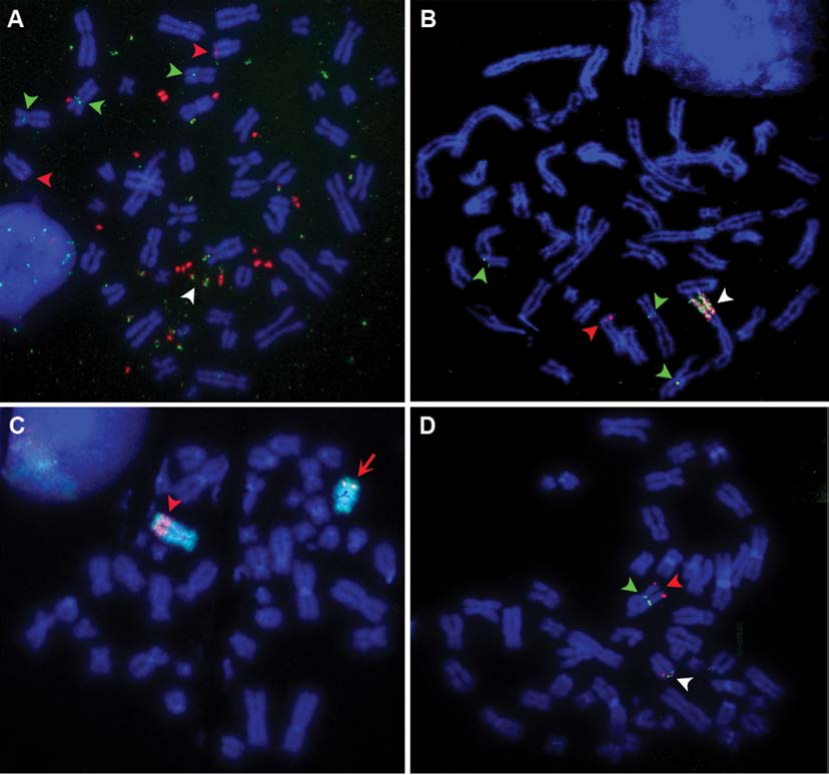
(**A**) ODA14p2. Co-hybridization of the BAC RP4–791C19 containing *EGFR* (green) and of the BAC RP11–1136L8 containing *MYC* (red). Dmins were labelled and co-hybridization of the probes was observed in some dmins (white arrow head). Two chromosomes 8 (red arrow heads) and 3 chromosomes 7 (green arrow heads) were labelled in 8q24 and 7p11, respectively. (**B**) ODA14p4. Co-hybridization of the BAC RP4–791C19 containing *EGFR* (green) and of the BAC RP11–1136L8 containing *MYC* (red). The hsr was labelled by the two probes (white arrow head). One chromosome 8 (red arrow head) and 3 chromosomes 7 (green arrow heads) were labelled in 8q24 and 7p11, respectively. (**C**) ODA14p4. Localization of the hsr on chromosome 8. Co-hybridization of the chromosome 8 painting (green) and of the BAC RP11–1136L8 containing *MYC* (red) showed that the hsr was inserted in a distal position of the long arm of chromosome 8 (arrow head). The *MYC* probe hybridized also on the normal chromosome 8 (arrow). (**D**) ODA14p4. Localization of the hsr on chromosome 8. RP11–69H6 (red arrow head) at position 129 328 282–129 517 488 and RP11–964F9 (green arrow head) at position 127 297 010–127 494 408 hybridized, respectively, in telomeric and centromeric positions of the hsr. The two BACs co-localized in 8q24 in the normal chromosome 8 (white arrow head).

The ODA14 tumour was selected after quantitative PCR screening of a series of gliomas for amplification of the *EGFR*,*MET*,*MYC* and *PDGFRA* genes, which are known to be recurrently amplified in these tumours (26,27). ODA14 displayed co-amplification of the *EGFR* and *MYC* genes at passage 2 (ODA14p2) and at passage 4 (ODA14p4). At both passages, pseudo-diploid cells with 47–49 chromosomes were observed. In ODA14p2, FISH using BAC overlapping the *EGFR* and *MYC* genes showed that they mainly localized to distinct dmins, although dmins containing both gene regions were also present (Figure 1A). More than 200 metaphases were analysed and amplifications were found in all cells and only on dmins, no hsr being detected. The 3 chromosomes 7 and the 2 chromosomes 8 present in these cells were labelled at 7p11 and 8q24, the normal locations of the *EGFR* and *MYC* genes, respectively (Figure 1A). At passage 4, one hsr containing entangled copies of the *EGFR* and *MYC* regions was observed (Figure 1B). More than 200 metaphases were also analysed and all cells contained the hsr, whereas cells containing dmins were not detected. In the following passages, up to passage 20, the hsr was present without modifications identifiable by FISH (not shown). Co-hybridization of the *MYC* gene with a chromosome 8-specific painting showed that the hsr maps to the distal position of the chromosome 8 long arm (Figure 1C). The 3 chromosomes 7 of ODA14p2 were still present in ODA14p4, but only one normal chromosome 8 remained (Figure 1C and D), which indicates that the hsr lies on the second chromosome 8. Further, FISH analysis localized the insertion in the 127.5–129.3 Mb region (Figure 1D). A more precise localization was not possible because of the overlap between the insertion region and the amplified segments (see below).

### Molecular characterization of the amplified regions

**Figure 2.**
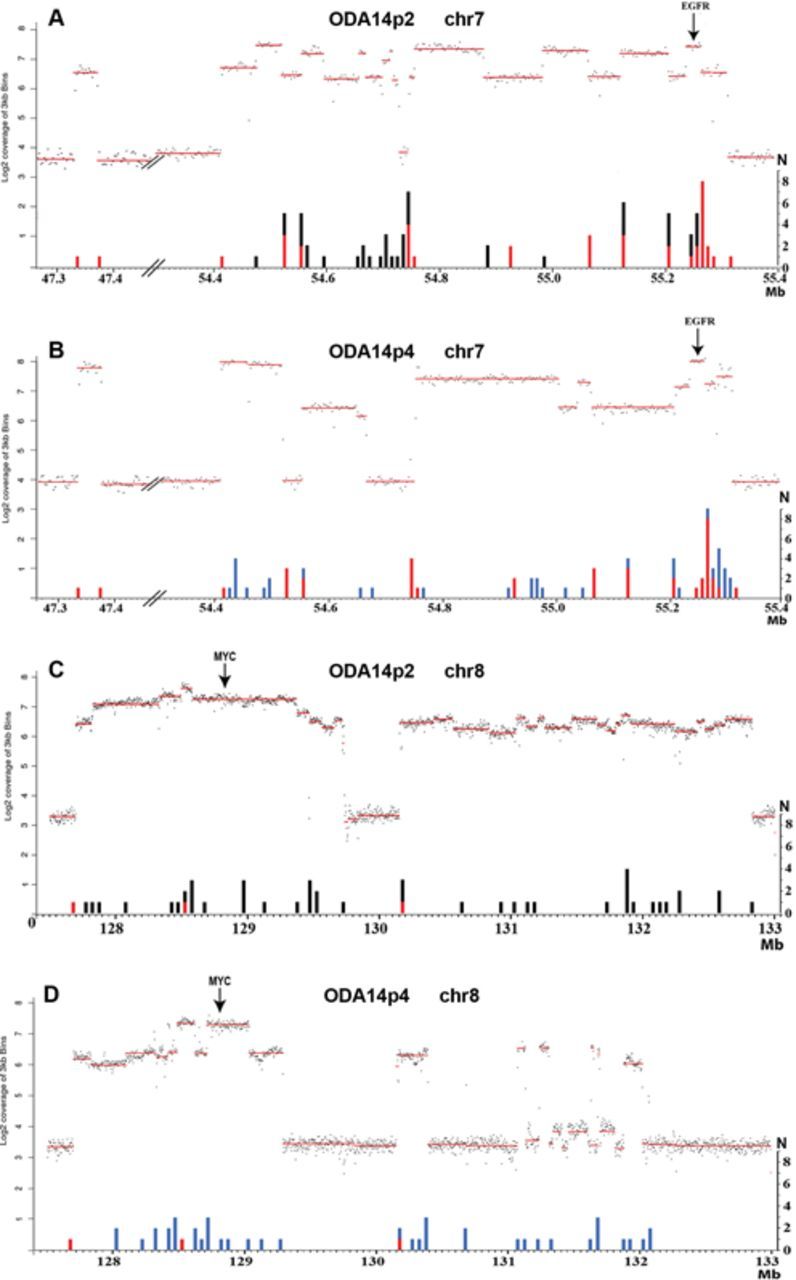
Genome sequencing coverage (3 kb bins) in the amplified regions of chromosomes 7 and 8 in ODA14p2 (A and C) and ODAp4 (B and D). The amplified regions are in dmins in ODA14p2 and in an hsr in ODA14p4. The log2 coverage profiles (upper parts, left scale) and the number of junctions (*N*) (lower part, right scale) are reported as a function of the position on the chromosome (in Mb). Three copies of chromosome 7 and two copies of chromosome 8 were present in the cells (Figure 1) corresponding to a coverage of the non-amplified regions near 4 and 3 for chromosome 7 and 8, respectively (see also Supplementary Data S1). Junctions were pooled by regions of 10 kb for chromosome 7 and 50 kb for chromosome 8. A and C: red, junctions remaining in ODA14p4; black, junctions not found in ODA14p4. B and D: red, junctions already present in ODA14p2; blue, new junctions.

The whole genome sequencing confirmed that the *EGFR* and *MYC* loci were amplified at both passages (Figure 2). No other loci, such as *MDM2* and *CDK4*, genes recurrently amplified in gliomas (28), were amplified in ODA14. Variations in the coverage were observed in the amplified regions. Roughly, two levels of amplification could be distinguished in series of segments, indicating that twice as many copies of some segments were present. However, lower variations were also present within these regions. In ODA14p4, the region 131.33–131.84 Mb of chromosome 8 was yet more complex with a series of low level amplified segments localized between unamplified and highly amplified segments. These data underline the presence of heterogeneities in the organization of the amplified regions. SNP analysis gave a similar overview of the amplified regions and indicated levels of amplification centred on 15-fold (Supplementary Data S1).

In ODA14p2, chromosome 7 sequences were amplified for a total of about 940 kb (Figure 2A) including 40 kb in the 47.3 Mb region and another segment localized between positions 54.41 and 55.31 Mb, which contain an unamplified region of about 20 kb in position 54.7 Mb. In ODA14p4, the 40 kb region remaining unchanged (Figure 2B). However, the second region displayed an altered pattern as compared with ODA14p2, with the loss of amplification for sequences in positions 54.52–54.56 Mb and 54.67–54.76 Mb. In ODA14p2, about 4.7 Mb of the chromosome 8 was amplified in two regions (from 127.70 to 129.73 Mb and from 130.15 to 132.82 Mb) (Figure 2C). Again, only sequences from these regions were amplified in the hsr of ODA14p4. The 5′ limit of the first amplified region remained unchanged but some 440 kb in 3′ were no longer amplified (Figure 2D). Only six segments corresponding to ∼20% of the second region remained amplified in ODA14p4 as compared with ODA14p2, but its 5′ limit was still present at 130.15 Mb (Figure 2D). Together, these results show that the hsr derived from the dmins. However, numerous rearrangements occurred during hsr formation and 15% and 55% of the sequences from chromosomes 7 and 8, respectively, amplified in dmins, were not integrated in the hsr.

### Identification of the junctions

**Figure 3.**
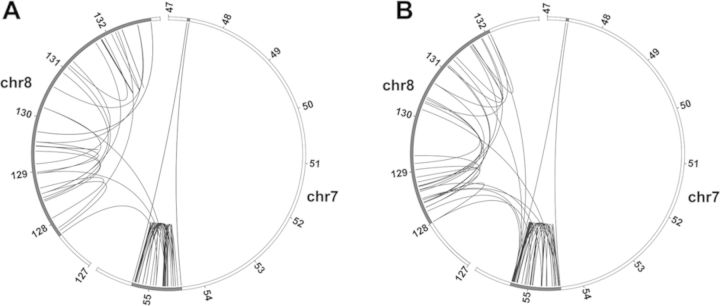
Circos plots of the position of the junctions in the amplified regions. (A) ODA14p2. B. ODA14p4. The amplified regions are in dmins in ODA14p2 and in an hsr in ODA14p4. Partial chromosome 7 and 8 regions are presented: the regions including the amplified sequences are in black. Only the junctions with the two arm sequences from the amplified regions are shown. Junctions were mainly between sequences from the same chromosome. A few interchromosomal junctions were present in ODA14p2 and their number increased in ODA14p4.

In order to obtain an overview of the rearrangements we searched for the presence of fusions between sequences non-contiguous in the reference genome. The whole genome sequence data of the tumour at both passages were used and junctions involving sequences localized in the amplified regions of chromosomes 7 and 8 were analysed (Supplementary Tables S1 and S2). A total of 105 junctions with at least one arm from these regions were identified in the dmins and/or the hsr. For 20 junctions, the presence of an insertion leading to the formation of additional junctions was observed and a total of 127 junctions were defined. Comparable numbers of junctions were found in ODA14p2 and ODA14p4 (65 and 62, respectively). Twenty-two junctions were present in both the dmins and the hsr and, finally, 105 distinct junctions were characterized (Supplementary Tables S1 and S2 and Supplementary Figure S1). In the dmins, the junctions occurred essentially between sequences from segments of either chromosome 7 or chromosome 8 (Figure 3A). However, the presence of three junctions associating sequences from chromosomes 7 and 8 (junctions 19, 20 and 28, Supplementary Table S1 and Figure 3A) indicated that fusions between the two amplified regions were already present. In the hsr, 43 junctions present in the dmins were lost and 40 new links between and within segments from chromosomes 7 and 8 were formed (Supplementary Table S2 and Figure 3B). Six junctions linked amplified sequences of chromosome 8 with sequences outside of the amplified regions of chromosomes 7 and 8. A 21-bp segment from the 114.48 Mb region of chromosome 8 associated the junctions 62 and 63. Four segments from chromosomes 3, 7 and 18 were also involved in the amplification process (junctions 61, 64, 65 and 105, Supplementary Tables S1B and S2B). Junction 61 was associated with an amplified segment of 1 kb in the chromosome 3, whereas no amplification was detected by sequencing or by SNP analysis at the other loci, suggesting that the amplified segments were very short. They were not further characterized. All junctions were confirmed at nucleotide resolution by re-sequencing except for junctions 64 and 65 (Supplementary Tables S1B and S2B), for which we failed to obtain a specific PCR product, likely because of the highly repetitive nature of the regions. These two junctions were excluded from the study. These sequencing data confirm that the hsr derived from the dmins and that new rearrangements, including the loss of segments amplified in dmins, occurred during the hsr formation.

### Junction characteristics

The positions of the two arms of a junction could be separated by Mb in the normal genome, whereas only 10% of them were less than 10 kb apart (including a single case below 1 kb), and in 11 cases the junctions were between sequences of chromosomes 7 and 8 (Supplementary Tables S1 and S2). The junctions 50, 60 and 57 in ODA14p2 and 84 in ODA14p4 resulted in the deletion of known structural variants (Supplementary Data S2).

More than half of the junctions took place in non-repeated sequences, the others occurring mainly in LINE, SINE and LTR repeats (Supplementary Table S3). No significant differences in these distributions existed between chromosomes 7 and 8 or between ODA14p2 and ODA14p4. The same interspersed repeated element was found on both sides of ∼10% of the junctions, but the fusion took place in non-homologous sequences.

**Figure 4.**
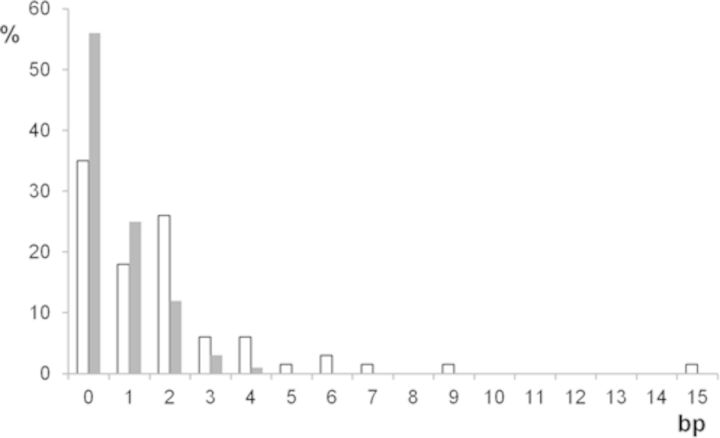
Distribution of the microhomology lengths at junctions. Filled bars**:** expected rate of microhomology if joining was random with respect to bp overlap; Empty bars: observed values.

No sequence homologies that could support a homologous recombination mechanism were found at the junctions (Supplementary Figure S1). Microhomologies, mainly of 1 or 2 bp, were present in the normal counterparts of the fusion and maintained as a single copy in 43% of the junctions. In 30% of cases, the junctions could not be aligned with the reference genome without the insertion of a short DNA sequence (Supplementary Tables S1 and S2). The insertions were generally below 20 bp in length and could not be localized in the reference genome. Among the six longer sequences (28–42 bp), partial homologies were found with other sequences of the genome in four cases (Supplementary Figure S2). We did not identify identical or imperfect copies of the flanking sequences that could indicate a templated insertion mechanism. Thus, these inserted sequences may represent non-templated DNA synthesis and/or ligations of very short DNA fragments. Considering only the junctions without insertions, 65% of the junctions contained microhomologies. This incidence of microhomology was significantly greater than that expected by chance, as only 44% of junctions would be expected to have microhomology if joining was random with respect to bp overlap (Figure 4). These results are consistent with a role for non-homology- and microhomology-mediated mechanisms in the formation of the junctions. Recently, a high rate of nucleotide variations and small deletions was observed in the vicinity of junctions in some rearrangements and attributed to the low processivity of the polymerase involved in replication-based mechanisms of break repair (18,29). In ODA14, no small deletions were localized in the 50-bp sequences both sides of the junctions and we identified only five transition or transversion events (G to A or G to C) at five junctions (2, 5, 18, 19 and 82; Supplementary Figure S1). None of these nucleotide variations was present in the dbSNP data base, suggesting that they do not represent common polymorphisms. This lack of a high rate of mutations in the vicinity of the junction was not in favour of the involvement of a replication-based mechanism in their formation. Finally, no significant differences in the junction characteristics were found between chromosomes or passages (Supplementary Figure S2).

### Localization of the breakpoints

The breakpoints involved in the junctions were distributed throughout the amplified regions (Figure 2). However, in chromosome 7, the positions of the breakpoints in the normal genome were recurrently only a few tens or hundreds of bp distant and tended to cluster. In ODA14p2, 12 clusters, ranging from 127 to 2497 bp, contained 3–7 breakpoints and five groups of two breakpoints could be listed (Table 1). These clusters put together 75% of the breakpoints (59/77). During the formation of the hsr of ODAp14p4, four new clusters containing 11 breakpoints were observed, whereas six new breakpoints were formed in four of the clusters observed in ODA14p2 (Table 2). With one exception in cluster O, the two breakpoints of a junction were not found in the same cluster (Tables 1 and 2). The clusters were recurrently localized close to large transitions in the DNA copy numbers. This relationship, however, was not exclusive since several transitions were associated with a single breakpoint (Figure 2). Breakpoints were less clustered in the chromosome 8 amplified regions where a single cluster of three breakpoints and three groups of two breakpoints were present in ODA14p2 corresponding to 21% (9/43) of the breakpoints (Supplementary Table S4). During the formation of the hsr, eight new groups of two breakpoints were formed (Supplementary Table S4). No sequence homology was found between the sequences of the clusters that did not contain particular repeated elements. Thus, hot spots for break formation were present in the amplified regions. Despite its shorter length, the amplified region of chromosome 7 had more clusters than the chromosome 8-amplified region and these clusters were more complex suggesting differences in the mechanisms of rearrangement.

**Table 1.** ODA14p2: clusters of breakpoints in chromosome 7

Cluster	Breakpoints (distance in bp)	Length	Position
A	a3 (141) a4 (1192) b12 (316) a5 (469) a6	2118	54 520 243
B	a7 (821) a8 (128) a9 (246) a10 (353) a11 (656) a12 (293) a13	2497	54 558 285
C	a16 (61) b11	61	54 668 755
D	a19 (36) a18 (91) b36	127	54 697 888
E	a21 (222) b2	222	54 738 501
F	a22 (1) a23 (41) b5 (17) b6 (34) a24 (10) a25 (265) a26	376	54 748 976
G	a27 (1823) a28	1823	54 880 905
H	a29 (244) a30	244	54 928 591
I	a31 (386) a32 (921) a33	1307	55 064 954
J	b15 (22) a34 (1792) b8 (306) b4 (108) b3 (40) a35	2227	55 123 258
K	b24 (290) b7	290	55 202 557
L	b14 (66) a36 (1461) b17	1527	55 206 654
M	a37 (30) b13 (160) b9	190	55 241 284
N	b23 (331) b33 (165) b35 (885) b21 (613) b26	1999	55 257 873
O	b39 (171) b32 (5) b25 (82) a38 (126) a39	384	55 262 169
P	b30 (329) b38 (505) b37	834	55 268 502
Q	b10 (348) b29 (1133) b31	1481	55 278 611

Each breakpoint is identified by the arm of the junction (Supplementary Table S1). The distance between breakpoints in the normal genome is given between brackets. The total length of the cluster and the position of the first breakpoint are indicated.

**Table 2.** ODA14p4: clusters of breakpoints in chromosome 7

7p4			
Cluster	Breakpoints (distance in bp)	Length	Position
B	a73	-	54 559 480
J	b83	-	55 125 302
O	a82	-	55 262 174
Q	a83, b73, b80	-	55 279 982–55 280 274
R	a78 (35) b77	35	54 957 228
S	b82 (103) a80	103	55 207 419
T	a67 (209) b68 (37) a69 (1109) b75	1355	54 438 342
U	a85 (11) b69 (19) b74	30	55 292 438

Legend in Table 1.

### Associations between segments

**Figure 5.**
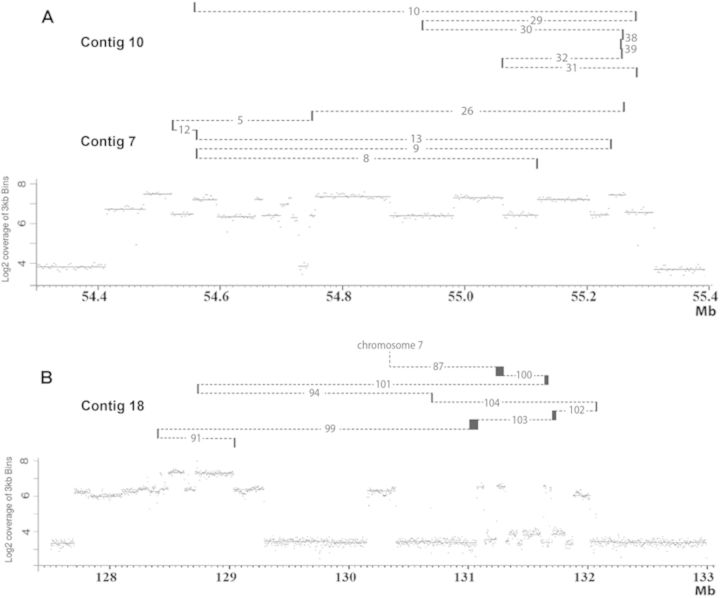
Structure of three contigs. (A) Contigs 7 and 10 (chromosome 7). (B) Contig 18 (chromosome 8). Lower parts: genome sequencing coverage in the amplified regions (see Figure 2). Upper parts: original position in the amplified regions of the segments included in the contigs. Small segments are localized by vertical bars and large segments of contig 18 by black boxes. Dotted lines visualize the junctions (see Table 3). Small fragments were generaly localized at the edge of copy number changes. However, fragments between junctions 102 and 104 and junctions 104 and 94 in contig 18 seems to come from non-amplified regions, but examination of the coverage at higher resolution showed that they were actually amplifed (Supplementary Figure S3).

In some cases, closely neighbouring sequences in the normal genome were involved in one arm of two different junctions (Supplementary Figures S1 and S2). To look for their possible association in a complex rearrangement, PCR between the neighbouring sequences of the two junctions was performed on the tumour DNA. Seventeen contigs were completely sequenced, including three corresponding to rearrangements common to ODA14p2 and ODA14p4 (Table 3). Eleven contigs contained two junctions, the others between 3 and 7 junctions. They comprised segments from the same chromosome, but in four cases where fragments from chromosomes 7 and 8 were associated (Supplementary Table S5). The segments included in the contigs contained 20–2118 bp (Table 3). In addition, in ODA14p4, a contig of eight segments with a total length of 150 kb was reconstituted (contig 18, Table 3). It comprised four large segments of 35, 14, 21 and 72 kb from the 131 Mb region (Figure 2D) and four small fragments of 0.2, 1.2, 1.1 and 4.3 kb localized outside of this region. As we failed to fully sequence the four large segments in the tumour DNA, we cannot exclude that undetected rearrangements escaped detection. However, no junctions other than the eight corresponding to these four large segments were present in this chromosome region (Figure 2D), suggesting that this 150-kb-long contig comprising eight reassociated fragments was present in the hsr. Further, chromosome walking from the ends of all contigs did not allow identification of other associated segments, likely indicating that the next associated segments were long. The contigs did not comprise segments that are neighbours in the normal genome (Figure 5 and Table 3). Hundreds of kb, up to several Mb, may separate the original positions of the small associated segments, excluding a mechanism of reassociation of fragments generated in the vicinity of an initial single break. In the contigs, blunt junctions were observed in 50% of cases and microhomologies higher than 2 bp were present in only 10% of cases (Table 3). Thus, during the amplification process, small and large fragments from non-neighbouring sequences participated in the formation of extra- and intrachromosomal amplified structures and no or very little microhomology was found at the junctions.

**Table 3.** Contigs observed in ODA14p2 and ODA14p4

Passage	Contig	Junctions	Microhomology (bp)	Length of fragments (bp)	Distance (kb)
p2	1	a11b - a16b	3 - 0	61	170
	2	a14b - a36b	0 - 0	66	105
	3	a15b - a34b	0 - 0	22	60
	4	a55b - a47b	0 - 0	28	815
	5	a2b - a21b - b23a	0 - 0 - 0	222 - 1386	782 - 10
	6	b18a - a19b - a53b	6 - 2 - 1	36 - 64	na - 1232
	7	b8a - a9b - b13a - a12b - a5b - a26b	2 - 1 - 1 - 0 - 0 - 0	128 - 160 - 293 - 316 - 326	116 - 1.5 - 719 - 188 - 737
					
p2 / p4	8	a24b - b7a	3 - 0	290	190
	9	a4b - b3a - a6b - a22b	2 - 1 - 0 - 4 -2 -2	108 - 2,118 - 67	0.14 - 376 - 235
		b31a - a32b			
	10	b39a - a38b - b30a - a29b -b10a	0 - 0 - 2 - 0 -5	386 - 171 - 126 - 329 - 244 - 348	17 - 197 - 6 - 333 - 10 - 369
p4	11	a73b - a83b - a35b	2 - 2 - 3	71 - 224	565 - 21
	12	a74b - a85b	0 - 2	30	na
	13	a75b - a69b	2 - 1	72	621
	14	a78b - a77b	2 - 0	35	12
	15	b92a- b98a	0 - 0	20	91
	16	a82b - a80b	0 - 0	109	18
	17	a88b - b81a	2 - 2	948	na
					
	18*	b91a - b99a - b103a -a102b -a104b - b94a - b101a - b100a - b87a	2 - 0 - 2 - 1 - 0 - 2 - 0 - 0	257 - 35 856 - 14 115 - 1258 - 1168 - 4331 - 21 471 - 72 238	2077 - 3297- 998 - 1000 - 1956 - 949 - 2588 - na

Junctions: junctions involved, a and b designate the two arms (Supplementary Tables S1 and S2). The microhomologies present at junctions and the length of each fragment, in bp, are indicated. Distance: distance between fragments in the normal genome (in kb); when a single fragment is present, this value corresponds to the distance between the breakpoint of each junction that does not border the fragment.

**Figure 6.**
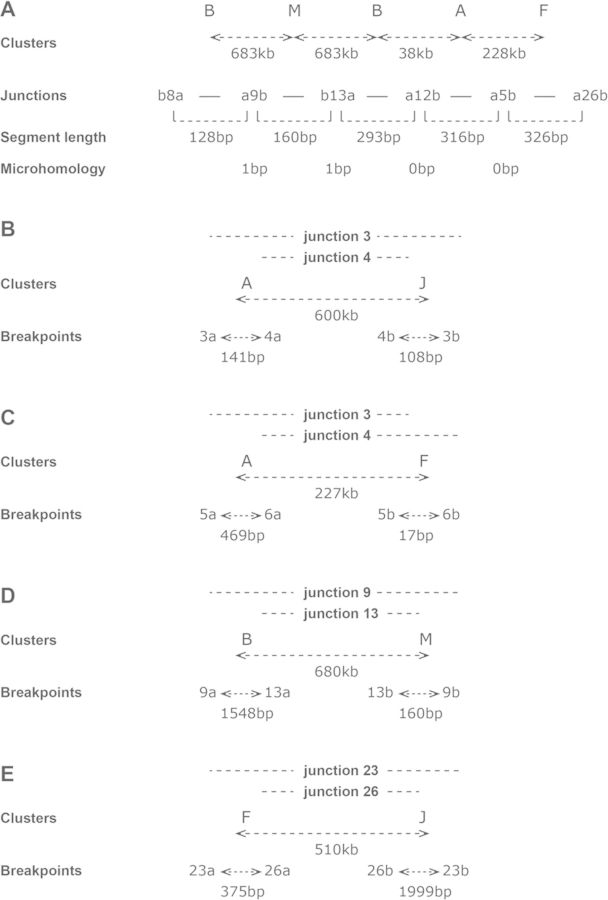
Concomitant formation of junctions in amplified regions of ODA14. Clusters are listed in Table 1, junctions are described in Supplementary Table S1 and a and b identify the two arms of the junctions. (A) Contig 7. The clusters that are tha source of the segments constituting the contig are indicated, with the distance between clusters in the normal genome. The length of segments and of the microhomologies at the junction are indicated. Two neighbouring segments from cluster B were associated with a segment from cluster M localized 683 kb away in the normal genome. This suggests that the chromosome regions of clusters B and M remained closely associated during the formation of the junctions. (B–E) Association between pairs of junctions. Clusters: clusters containing the breakpoints and distance between the clusters in the normal genome. Breakpoints: distance between the breakpoints in the clusters. This indicates that sequences localized hundreds of kb apart were put together and that the formation of each pair of junctions was concomitant.

A few situations suggested that at least some of junctions were not formed by association of free fragments. In contig 7 (Figure 6A), two neighbouring segments from cluster B were associated with a segment from cluster M localized 683 kb apart in the normal genome (Table 1). This suggests that the chromosome regions of clusters B and M remained closely associated during the formation of the junctions. Some particular breakpoint/junction associations reinforced this suggestion (Figure 6B). In these four cases, the arms of each junction came from clusters several hundred kb apart in the normal genome, whereas, in each cluster, breakpoints were separated by a few hundred of bp. This indicates that sequences localized hundreds of kb apart were put together and that the formation of each pair of junctions was concomitant. These junctions were not different from the others, with microhomologies of mainly 0 or 1 bp, and a single 2 bp microhomology (Table 3). Thus, it can be concluded that mechanisms using no or very short microhomologies prevailed in the formation of the contig and of the concomitantly formed junctions.

## DISCUSSION

In the xenografted human oligodendroglioma ODA14, the *EGFR* (7p11) and the *MYC* (8q24) loci were co-amplified and the amplified sequences resided on dmins at passage 2 (ODA14p2) and on an hsr at passage 4 (ODA14p4). The analysis by FISH failed to underline cells containing both dmins and the hsr.

No deletion was observed in the original intrachromosomal positions of the amplified regions of chromosomes 7 and 8, and it can be proposed that the amplification process was initiated by post-replicative events leading to the formation of initial extrachromosomal DNA molecules from the loci on chromosomes 7 and 8 (1). In both cases, a unique initial event was also suggested by the presence of common structures in all dmins, such as the unamplified segment localized between amplified segments in both chromosomes (Figure 2A and C). This suggests that rearrangements of the amplified region were formed during the initial formation of extrachromosomal DNA molecules. It cannot be established if the formation of the initial extrachromosomal DNA molecules from chromosomes 7 and 8 were formed concomitantly or were independent events. The sequences amplified in ODA14p2 display a complex organization, characterized by the presence of numerous segments with various DNA copy number levels (Figure 2 and Supplementary Data S1). The numerous junctions corresponding to fusions between non-contiguous sequences in the normal reference genome confirmed the presence of rearrangements in the dmins. These junctions essentially involved segments amplified regions from either chromosome 7 or chromosome 8. Nevertheless, a few junctions associating sequences from chromosomes 7 and 8 indicated that fusions between the two amplified regions were already present. These data show that differences in the association between segments were present within and/or between dmins as a consequence of rearrangements undergone after the initial event.

The site of insertion of the hsr overlapped the *MYC* amplified region and it was not possible to establish whether integration was associated with NHEJ mechanisms as recently shown (16) or involved homologous recombination between sequences of the *MYC* locus. However, the presence of the chromosome 8 distal region in the telomeric position of the hsr suggests that a breakage–fusion–bridge mechanism was not involved (30). As for dmins, a complex structure was observed for the hsr corresponding to heterogeneities in the segment copy number. The presence of numerous identical junctions in ODA14p2 and ODA14p4 showed that the hsr originated in the dmins. The formation of hsr by integration of dmins is well documented (9–13) and, in the few cases analysed in detail, no modification was observed in the extent of the amplified regions (5,8). In contrast, only 85% and 45% of the sequences of chromosomes 7 and 8 amplified in dmins were, respectively, found in the hsr. Simultaneously, junctions present in the dmins were lost, whereas numerous junctions not observed in the dmins were now present and new rearrangements between and within segments from chromosomes 7 and 8 were formed (Figures 2 and 3). This is particularly obvious for chromosome 8 where only three junctions were common to the two forms of amplification. Thus, the data suggest that the new rearrangements observed were not already present in a subset of dmins giving rise to the hsr and that the formation of the hsr was associated with massive formation of breakages leading to a new organization of the amplified sequences.

The complexity of the amplified regions prevented us from proposing for dmins and for the hsr credible global assemblies based on the connection of the segments involved in the junctions. However, the available data allowed us to investigate some of the mechanisms of amplification. It is noteworthy that no significant difference was found in the junction's characteristic between dmins and hsr, suggesting identical mechanisms.

The junctions without inserted nucleotides disclosed microhomologies, mainly of 1 or 2 bp, at a higher rate than expected by chance (Figure 4). These data suggest that mechanisms using microhomologies play a role in junction formation in agreement with previous data (1–3,5–8,14,15,31). When microhomologies <5 bp are used for alignment, NHEJ can be involved, whereas for longer microhomologies, an alternative mechanism (MMEJ) may predominate (32,33). In fact, it is difficult to distinguish the respective contributions of these two types of mechanisms because both can use short microhomologous sequences. However, the insertions of sequences that may come from non-templated synthesis or from the fusion of short fragments was observed in 60% of the junctions without microhomology, showing that the mechanisms of fusion may be more complex than proposed by present models.

Break-induced replication (BIR)-derived models were also proposed for the rearrangements that lead to junctions containing microhomologies. BIR is a pathway of homologous recombination that contributes to the repair of broken replication forks (reviewed in (34)). BIR is initiated by invasion of a single strand into a homologous DNA molecule followed by DNA synthesis, which may continue as far as the next replication fork or switch to homologous DNA templates. Replicative mechanisms with similarities to BIR, such as FoSTeS and MMBIR, have been proposed to explain the formation of complex genomic rearrangements and copy number variation (35,36). These rearrangements are initiated by strand breaks such as broken replication forks and involve extensive DNA synthesis and frequent template switches. The main difference between BIR and these mechanisms is that the template switching is not mediated by homologous recombination but relies on the presence of microhomologies. The involvement of a replication-based mechanism of amplification is thus possible in the ODA14. However, it has been shown that BIR mechanisms are extremely mutagenic with the frequent formation of point mutations and frameshifts in the synthesized segments, likely due to a low fidelity of the replication (18,29). No frameshift mutations were present near the junctions and few potential point mutations were observed. Finally, we did not identify the templated insertions of nearby sequences commonly observed in association with replication-based rearrangement mechanisms. Thus, it appears that NHEJ/MMEJ processes are more likely to be involved as it is difficult to reconcile the BIR-derived models with the massive reassociation of small segments we detected in ODA14 dmins and hsr.

Thus, our data are more in line with models based on chromosome fragmentation (shattering, pulverization, chromothripsis), where chromosomes are under replication stress leading to DNA double-strand breaks and to the formation of numerous fragments (reviewed in (37,38)). The process may be limited to one or a few chromosomes possibly after their trapping in micronuclei (39). These fragments, possibly localized hundreds of kb apart or on different chromosomes in the normal genome, are further randomly associated in complex structures. However, some specific characteristics suggest that, in ODA14, the processes of breakage and fusion were not necessarily random.

Both in the dmins and in the hsr, the amplifications comprised a mix of large and small fragments. The insertion of small fragments (‘genomic shards’) at breakpoints between large chromosome segments was previously observed in cancer cells (14,15). These fragments were usually tightly clustered within a few kb of each other and it was proposed that small DNA fragments were generated in the vicinity of a chromosome break, by either physical or enzymatic processes, and subsequently fused. In ODA14, the small fragments came from chromosomal regions hundreds of kb apart. Moreover, in contig 18, where association between four small and four large fragments was characterized, the large segments were clustered in a region of 0.6 Mb and the narrowest sequence involved in the small fragments was 374 kb apart indicating that the breakpoints leading to large fragments did not drive the linkage of the small fragments. However, these small fragments came from different clusters of breakpoints distributed over a few hundred or thousand bp. This clustering suggests a perturbation of the replication process. It has been proposed that slippage events in the replication fork could lead to such clustering (18). However, this mechanism was unlikely in ODA14 since junctions were not established between the neighbouring breakpoints of a cluster, but with distant sequences.

No similarities between sequences were found in the cluster regions that could suggest a mechanism leading to the formation of these hot spots of breakage. It can be assumed that these small fragments were the product of an unstable process that involves attempts to reform the replication fork in a locus difficult to replicate. Not all breakpoints of a cluster were necessarily generated together during a single replication, but could arise in different amplicons during successive steps of the amplification process, as shown by the formation of new breakpoints in four clusters during the dmins/hsr transition (Table 2). Breakpoint clustering capacity seems to differ in the amplified regions of chromosome 7 and 8: in the dmins, the clusters put together 75% of the chromosome 7 breakpoints and only 21% of those of chromosome 8. In association with other differences (fewer breakpoints in chromosome 8 than in chromosome 7, loss of a large fraction of the amplified regions of chromosome 8 during the dmins/hsr transition whereas, in chromosome 7, only a small fraction was lost), these data suggest that the replication process differs in the two regions. The origin of these differences remains an open question, but it can be noted that two replication stress-inducible common fragile sites are present in the *MYC* gene locus (FRA8C and FRA8D (40)), whereas no fragile site is known in the region surrounding the *EGFR* gene (41). The differences in the replication process in common fragile sites as compared with the other parts of the genome (42,43) could play a role in the final structure of the amplified sequences. Characterization of the amplifications from these and other regions will be necessary to confirm that the amplification mechanism can vary depending on the genome location.

Generally, there seems to be no rule governing the choice of the fragments to be associated, but particular situations were observed. In contig 7, two fragments from a cluster were linked both sides of a fragment from another cluster localized at 683 kb from the first (Figure 6A). This structure could correspond to a template switch between the two clusters, but the microhomology of a single base at the junctions that may have occurred by chance is not in favour of such a mechanism. Nevertheless, coordinated repair of the double-strand breaks can be proposed where the chromosome regions of clusters B and M remained closely associated during the formation of the junctions.

Indices of coordinated events were also present in other junctions. In four cases, two closely neighbouring breakpoints were linked to two other closely neighbouring breakpoints localized hundreds of kb apart (Figure 6). The presence of these particular fragment organizations suggests that, at least in some cases, the process was not a fusion of free small fragments but rather a coordinated mechanism where distant regions were moved closer together in a complex allowing the coordinated repair of the double-strand breaks and the formation of the junctions. The possibility of associations between remote sequences has been previously demonstrated (44,45) and it has recently been shown that the V(D)J recombination leads to the rapid association between sequences separated by Mb (46). It is noteworthy that a V(D)J-like rearrangement was present in a dmins present in a glioma (3), suggesting that such long-distance interactions are possible during the amplification process. The nature of the mechanisms remain to be established, however, the lack of microhomologies with more than 2 bp suggests that BIR-derived models are unlikely and that NHEJ/MMEJ processes were involved.A main difference between the literature data and ours is the presence in the amplified sequences of numerous small non-contiguous segments associated in contigs. Such organization was observed only in three cell lines containing hsr (14,15) and was not found in several other whole genome sequencing analyses of tumours and established cell lines (2,5–8). It is remarkable that the studied cell lines harboured stable dmins whereas, in our case, the dmins present at early passages of the xenograph were rapidly replaced by an hsr, showing their instability outside of the original tumour context. Thus, it can be assumed that, depending on the specific characteristics of the original tumour, different genome instability mechanisms may lead to extrachromosomal amplification and that stable and unstable dmins are formed and evolve using different pathways.

Xenografted human tumours allow us to use 3D tumours, which are considered as more like original tumours than cells growing *in vitro*. It was thus possible to characterize the amplification process with a precision not possible when independent clonal cell lines are used. One limitation of the approach is that xenografts that grow well must be obtained, a situation found in only a minority of cases. With ODA14, despite poor growing growth, two early xenografted passages containing dmins were obtained, a case rarely found even when dmins are present in the original tumour. After passage 4, cells containing the hsr grew easily. The xenografts were maintained up to passage 20 without changes in the cytogenetic appearance of the hsr. The molecular analysis of these late passages was not performed but a possible instability of the sequence organization in the hsr could be studied by sequencing. Passage 3, corresponding to the transition between dmins and hsr, would have been very interesting to study. Unfortunatly, at this passage a considerable cell mortality was observed and it was assume that only the cellular clone with the hsr was growing. Thus, the few available cells were used for the xenograft. Due to this limitation, even *in situ* analysis was not possible and the formation of the hsr could not be monitored.

In conclusion, this characterization at nucleotide resolution of the transition between extra- and intrachromosome amplifications highlights a hitherto uncharacterized organization of the amplified regions suggesting the involvement of new mechanisms in their formation. The detailed characterization of other examples of the evolution of the amplified forms in cancer cells will be necessary to establish their actual recurrence and to show if different pathways are involved in cancers during the amplification processes.

## ACCESSION NUMBERS

Sequence data were recorded in the Sequence Read Archive (SRA) database (Accession: ODA14p4, SRS476246; ODA14p2, SRS476247).

## SUPPLEMENTARY DATA

Supplementary Data available at NAR Online.

SUPPLEMENTARY DATA
