# Supplementary material for: Amplicon rearrangements during the extrachromosomal and intrachromosomal amplification process in a glioma
Source: Nucleic Acids Res. 2014 Nov 6;42(21):13194–205. doi: 10.1093/nar/gku1101 (PMC4245956; doi:10.1093/nar/gku1101)
Supplement: SUPPLEMENTARY DATA [file supp_42_21_13194__index.html]

Amplicon rearrangements during the extrachromosomal and intrachromosomal amplification process in a glioma — Amplicon rearrangements during the extrachromosomal and intrachromosomal amplification process in a glioma — SUPPLEMENTARY DATA 

# Amplicon rearrangements during the extrachromosomal and intrachromosomal amplification process in a glioma

## SUPPLEMENTARY DATA

**Files in this Data Supplement:**

- SUPPLEMENTARY DATA
