# Supplementary material for: Amplicon rearrangements during the extrachromosomal and intrachromosomal amplification process in a glioma
Source: Nucleic Acids Res. 2014 Nov 6;42(21):13194–205. doi: 10.1093/nar/gku1101 (PMC4245956; doi:10.1093/nar/gku1101)
Supplement: SUPPLEMENTARY DATA [file supp_gku1101_nar-02726-z-2014-File011.pdf]

Table S1

A

Junction	strand	arm a chro 7	chro 8	strand	arm b chro 7	chro 8	Distance between arms	Microhomology	insertion	mapping number
1	+	47377914		+	54414157		7,036,243	1		18
2	-	54476685		-	54738723		262,032		3	15
3	-	54520243		-	55125486		605,243	1		8
4	+	54520384		+	55125378		604,994	2		6
5	+	54521892		+	54749026		227,134			6
6	+	54522361		-	54749043		226,682			4
7	-	54558285		-	55202847		644,562		36	4
8	-	54559106		+	55125072		565,966	2		12
9	+	54559234		-	55241474		682,240	1		3
10	-	54559480		+	55278611		719,231	5		8
11	+	54559833		-	54668816		108,983	3		4
12	-	54560489		+	54521576		38,903		3	13
13	+	54560782		+	55241314		680,532	1		5
14	+	54595424		+	55206654		611,830		3	7
15	-	54659764		+	55123258		463,494		14	8
16	-	54668755		-	54730348		61,593			4
17	+	54671909		-	55208181		536,272	2		9
18	+	54697888		-	54701077		3,189	6		4
19	-	54701041		+		130190336	na	2		4
20	-	54713621		+		128958218	na			13
21	-	54738501		-	55259259		520,758			12
22	-	54748976		+	54758053		9,077	4		4
23	-	54748977		+	55257873		502,896	1		11
24	-	54749077		+	55202557		453,480	3		16
25	-	54749087		-	55262345		513,258	2		11
26	+	54749352		+	55259872		510,520		1	11
27	+	54880905		+	54986016		105,101		28	6
28	+	54882728		+		127757114	na		1	3
29	-	54928591		-	55278959		333,911			6
30	+	54928835		+	55268502		345,667	2		10
31	-	55064954		+	55280092		215,138	2		11
32	+	55065340		+	55262340		197,000	2		10
33	+	55066261		+	55258204		192,943			6
34	+	55123280		-	54720579		402,701			8
35	+	55125526		-	55258369		132,843	3		11
36	+	55206720		+	54701168		505,552		1	7
37	-	55241284		-	55269336		280,052			14
38	+	55262427		-	55268831		6,404			5
39	+	55262553		+	55262169		384			5
40	+	55315470		+	47338022		7,977,448	3		13
41	-		127825883	-		128579780	753,897		7	7
42	-		127878972	-		132198438	4,319,466		5	4
43	-		128093657	-		128687565	593,908	7		3
44	+		128433071	-		129473548	40,477			4
45	-		128460114	-		131055616	2,595,502	1		4
46	-		128503400	-		129374147	870,747		1	3
47	+		128579178	-		131739197	3,160,019			3
48	+		128984176	-		129545398	561,222		1	3
49	+		129139899	+		131199290	1,734,133			6
50	+		129465157	+		129471271	6,114	15		28
51	+		129546684	+		132578151	3,031,467		9	4
52	+		129735150	-		132827500	3,092,350		7	19
53	+		130190400	-		128957699	1,232,701	1		4
54	-		130612941	+		132581669	1,968,728	2		6
55	-		130923423	+		128579150	2,344,273		7	3
56	+		131104145	-		131928335	824,190	2		4
57	+		131850749	+		131852739	1,990		1	12
58	-		131862826	+		131868801	5,975			3
59	+		132071238	+		132148229	76,991			3
60	+		132277696	+		132280638	2,942			6

B

Junction	arm a chromosome, strand	arm a position	arm b chromosome, strand	arm b position	Microhomology	insertion	mapping number
61	chr3 -	111274086	chr8 +	128533830			29
62	chr8 -	130152.661	chr8 +	114484890			16
63	chr8 +	114484910	chr8 +	127698232			16
64	chr7 +	682390	chr8 +	127865503			5
65	chr3 +	29048987	chr8 -	131018608			3

Table S2

A

Junction	strand	arm a chro 7	chro 8	strand	arm b chro 7	chro 8	Distance between arms	Microhomology	insertion	mapping number
1	+	47377914		+	54414157		7,036,243	1		33
66	-	54427305		-	54918735		491,430	4		4
67	-	54438342		+	55044062		605,720		11	22
68	+	54438551		+	54490561		52,030		1	3
69	-	54439588		+	55292449		852,801	1		5
70	+	54451802		+		128484872	na	6		8
71	+	54488433		+	54966885		457,452		18	3
72	+	54496942		-		130152776	na			3
3	-	54520243		-	55125486		605,243	1		8
4	+	54520384		+	55125378		604,994	2		10
6	+	54522361		-	54749043		226,682			25
7	-	54558285		-	55202847		644,562		36	10
10	-	54559480		+	55278611		719,131	5		15
73	+	54559825		+	55280053		720,228	2		4
74	+	54657308		-	55292468		615,160		4	6
75	+	54670874		-	54439660		231,214	2		5
22	-	54748976		+	54758053		9,077	4		25
24	-	54749077		+	55202557		453,480	3		16
25	-	54749087		-	55262345		513,258	2		12
76	+	54765765		+		128648771	na		2	6
29	-	54928591		-	55278959		339,911			10
30	+	54928835		+	55268502		345,667	2		12
77	+	54957228		-	54973297		16,069		8	4
78	+	54960833		+	54957193		3,640		2	4
79	+	55011056		-		128016981	na		10	4
31	-	55064954		+	55280092		215,138	2		10
32	+	55065340		+	55262340		197,000	2		9
33	+	55066261		+	55258204		191,945			11
35	+	55125526		-	55258369		132,843	3		9
80	+	55207522		-	55280274		72,752		2	3
81	-	55216848		-		128458972	na	2		15
37	-	55241284		-	55269336		280,052			30
82	-	55262174		+	55207419		54,755			3
38	+	55262427		-	55268831		6,404			5
39	+	55262553		+	55262169		384			5
83	-	55279982		-	55125302		154,680	1		4
84	+	55286758		+	55289062		2,304	15		24
85	-	55292438		+		128233714	na	2		6
86	-	55301671		+		132022399	na	1		16
87	-	55306136		+		131240408	na		37	13
40	+	55315470		+	47338022		7,977,448	3		54
88	-		128016842	+		128458024	441,182	2		3
89	-		128310905	-		130393312	2,088,407	9		10
90	+		128314386	-		129289585	975,199	1		16
91	+		128401487	-		129032908	631,421	2		19
92	+		128620473	-		130361150	1,740,677		42	4
93	+		128656514	+		131883074	3,226,560		3	16
94	+		128728355	+		130683305	1,954,950	2		13
95	-		128800883	-		128890626	510,257		37	3
96	-		129147354	-		130282672	1,135,518		2	3
97	-		130313622	+		131901429	1,587,807	4		5
98	-		130361130	+		128711699	1,649,431		13	4
99	+		131110042	+		128401250	2,708,792		3	11
100	+		131312646	-		131654660	342,014		30	6
101	-		131633189	+		128724024	2,909,165			17
102	-		131684708	-		132072532	387,824	1		9
103	-		131698823	+		131074186	624,637	2		18
104	-		132071274	-		130684473	1,386,801		2	23

B

Junction	arm a chromosome, strand, position		arm b chromosome, strand, position		Microhomology	insertion	mapping number
61	chr3 -	111274086	chro8+	128533830			29
62	chro8 -	130152661	chr8 +	114484890			18
63	chr8 +	114484910	chro8+	127698232			18
105	chr18 -	73918393	chro8-	131412731			4

Table S3

A

Repeat at junction	chrom 7	chrom 8
no	48.4	48.9
LINE	23.4	18.1
SINE	11.7	17.7
LTR	11.7	13.6
DNA	3.9	1.5
Low complexity	0.7	-

B

Repeat at junction	ODA14p2	ODA14p4
no	51.3	46.1
LINE	24.3	20
SINE	10.8	16.3
LTR	13.5	11.6
DNA	-	5
Low complexity	-	0.8

C

Repeat at junction	Number	Percentage
no	108	51
LINE	42	20
SINE	30	14.2
LTR	23	10.9
DNA	6	2.8
Low complexity	1	0.5

Table S4 chromosome 8

Cluster	Passage	Breakpoints (distance in bp)	Length	Position
A'	p2	b55 (28) a47 (602) b41	630	128,579,178
B'	p2	b53 (517) b20	517	128,957,699
C'	p2	b48 (1486) a51	1486	129,545,398
D'	p2	a53 (64)b19	64	130,190,336
E'	p4	a88 (139) b79	139	128,016,842
F'	p4	b99 (237) a91	237	128,401,250
G'	p4	b88 (948) b81	948	128,458,972
H'	p4	b101 (4331) a94	4331	128,724,024
I'	p4	a62 (115) b72	115	130,152,661
J'	p4	a98 (20) b92	20	130,036,113
K'	p4	b94 (1168) b104	1168	130,683,305
L'	p4	a104 (1258) b102	1258	132,071,274

Table S5

Passage	Contig	Junctions	Chromosomes	Length (bp)
p2	1	- 11 - 16 -	7 - 7 - 7	61
	2	- 14 - 36 -	7 - 7 - 7	66
	3	- 15 - 34 -	7 - 7 - 7	22
	4	- 55 - 47 -	8 - 8 - 8	28
	5	- 2 - 21 - 23 -	7 - 7 - 7 - 7	1,608
	6	- 18 - 19 - 53 -	7 - 7 - 8 - 8	100
	7	- 8 - 9 - 13 - 12 - 5 - 26 -	7 - 7 - 7 - 7 - 7 -7	1,223
p2 and p4	8	- 24 - 7 -	7 - 7 - 7	290
	9	- 4 - 3 - 6 - 22 -	7 - 7 - 7 - 7 - 7	2,293
	10	- 31 -32 - 39 - 38 - 30 - 29 - 10 -	7 - 7 - 7 - 7 - 7 - 7 - 7 - 7	1,603
p4	11	- 73 - 83 - 35 -	7 - 7 - 7 - 7	295
	12	- 74 - 85 -	7 - 7 - 8	30
	13	- 75 - 69 -	7 - 7 - 7	72
	14	- 78 - 77 -	7 - 7 - 7	35
	15	- 92 - 98 -	8 - 8 - 8	20
	16	- 82 - 80 -	7 - 7 - 7	109
	17	- 88 - 81 -	8 - 8 - 7	948
	18*	- 91 - 99 - 103 -102 -104 - 94 - 101 -100 - 87 -	8 - 8 - 8 - 8 - 8 - 8 - 8 - 8 - 7	150,694

Figure S1

Sequences of the junctions

1
(7) 47377914 | 54414157 (7)
ACAGCAGCTGTACCATTTTAACAATCCCTCCAGCAACGTATAAGGGGTCTAGTTTTTCCACATGCTCACCAGT
ACAGCAGCTGTACCATTTTAACAATCCCTCCAGCAAGCCAAGGTTTTGCCATTGCACTCCAGCCTGGGTGACA
GAATCACTTGAACCCGGGAGGCAGAGGTTGCAGTGAGCCAAGGTTTTGCCATTGCACTCCAGCCTGGGTGACA

2
(7) 54476685 | 54738723 (7)
TGGTAAATGAAGAATTAGTACTAAAGTCAACTATGAGATAATGAAATTTATCTTGAGATTGGCATATGATGATTG
TGCTAAATGAAGAATTAGTACTAAAGTCAACTATGAGAGAAAGAAGAGGTGCTTTTCTTTCTGTATTTTTTATTA
TACTAAACTGTACAGGAAAATCCATGGTCCAAAAGTATAGGAGAAGAGGTGCTTTTCTTTCTGTATTTTTTATTA

3
(7) 54520243 | 55125486 (7)
ACTGAGCATTTTCAATTAACTGTGTGGACTGAACAACATATGAAGAATCAGAAATCACATTAATAGGCATATCAAAGG
ACTGAGCATTTTCAATTAACTGTGTGGACTGAACAACATATAAATGTGAGAACAATACCGCAGAGATAATGAGCTGTC
TCTTCAGGCCTGAGAGGCCTCTGCCTCCGCTAACCTCACTTAAATGTGAGAACAATACCGCAGAGATAATGAGCTGTC

4
(7) 54520384 | 55125378 (7)
GAAGTAGAATGCACCCTTTTTACGTGACTCACATTAGAGCTCATACACTCTTCCAGGACCTTTGACTGAAGGAAATC
GAAGTAGAATGCACCCTTTTTACGTGACTCACATTAGAGCTTGATTTTCTAACATAAAATGGGATTGAGAGGGGAAT
CTTGTTTTGGTCACATAATTTAACTTAACATTCTCGGTACTTGATTTTCTAACATAAAATGGGATTGAGAGGGGAAT

5
(7) 54521892 |54749026 (7)
ACAAAACGGTCAGATGGTAGAAATGATAAGAAGTACAGCAAAAGTTCCTATTATCTGGAACCATGGTGTTACAGT
ACAAAACGGTCAGATAGTAGAAATGATAAGAAGTACAGTGGTTCAATCCAATGGTTATTTCTCAGTCTTAACTGA
AGGTTCTGGTCTTCCCTACGACATCAGTGACCTTCATTTGGTTCAATCCAATGGTTATTTCTCAGTCTTAACTGA

6
(7) 54522361 | 54749043 (7)
CCCTTGCTTTTATCTCTTTGCAAATCAAAGAAGGGAGACGTGTTGGAAGCAAGCCCCCCTAAAATCTGGCCATAAACT
CCCTTGCTTTTATCTCTTTGCAAATCAAAGAAGGGAGACGAACCATTGGATTGAACCAAATGAAGGTCACTGATGTCG
ATAAATGGCAAGCCAAAAATTTCAGTTAAGACTGAGAAATAACCATTGGATTGAACCAAATGAAGGTCACTGATGTCG

7
(7) 54558285 |55202847 (7)
TTGGGAGATGATTAGGTCATGAGACAGAATCTTCATAAATGGGATGAGCACCCTTATAAAAAAGGCCTGTTATACAT
TTGGGAGATGATTAGGTCATGAGACCTAACATATGCATAACAGATGAGTGGGAAAGAAACCATTTGTAAAATATACA
TGGACTTCCAACCCCATGATTCTCCTTCTAGCCATTAAAAGAATCCAAAGCCAAGCTTGTGATTTGTAAAATATACA

8

(7) 54559106 | 55125072 (7)

GCATGATCCTTAATTGCATTTTCTGCAGAAAAATTATGATGAATGTGCTCTTACATTGCAATGCGAAATACGGTA
GCATGATCCTTAATTGCATTTTCTGCAGAAAAATTATCTCATAAGTGAACAAAACCCAGCCCTTCAAAGAAGTCA
GGACTTCCCTGACACTGAGCACCTCTTAATTAAGCATCTCATAAGTGAACAAAACCCAGCCCTTCAAAGAAGTCA

9

(7) 54559234 | 55241474 (7)

GATATATAATGGTTGTGCAATGCTTTGACAATGATTGTCGAGACTGTTAATTTCACTGTCTCAGTATAACAGGGA
GATATATAATGGTTGTGCAATGCTTTGACAATGATTGTTGGAAGGACAAATGTACGCCTTCTCTACAGAGCTTGA
GGGCTTGGGTGCCAGGACACGGCACTTGCCAGCTCATTTGGAAGGACAAATGTACGCCTTCTCTACAGAGCTTGA

10

(7) 54559480 | 55278611 (7)

AATGCGCCCAGGAAAACAAGCAGGAACAGAAAGCAAGCAGAACAGAGGAAGTCCGTGCTCTCTCTTCTTCCAGGATCC
AATGCGCCCAGGAAAACAAGCAGGAACAGAAAGCAAGCAGATTTCAAGAGGATTGAGTAAGTAGTTGGATGGCTTTCA
AAAGAAAACGCTGGCCTATCAGTTACATTACAAAAGGCAGATTTCAAGAGGATTGAGTAAGTAGTTGGATGGCTTTCA

11

(7) 54559833 | 54668816 (7)

ATAAAGGTGTTCTCGCAGGTGTGCCTTCCTGACACCTTAACCTCCTATTTTTTAGTCTCTAAAACCCAGTTAAAA
ATAAAGGTGTTCTCGCAGGTGTGCCTTCCTGACACCTTCCATCTAGCCTTTTCTACTGGATTGGTGTCTAATTTA
GGTGAGGCCAGTGGGAAGGGAAGCATTTCCAGGGGCTTCCATCTAGCCTTTTCTACTGGATTGGTGTCTAATTTA

12

(7) 54560489 | 54521576 (7)

TAGAGAGACAAGGTCTCACTCCATCGCCCAGGCTGGAGTGCAATGGCAATGACATGATTATAGTTCACTGCAGTC
TAGAGAGACAAGGTCTCACTCCATCGCCCAGGCTGGAGATACAATCACTTTATGACACAGTTACACATGCTTTCT
AGGTATATTTATCAATCTTGGCTGGCAATAATGCCTGGATGCAATCACTTTATGACACAGTTACACATGCTTTCT

13

(7) 54560782 | 55241314 (7)

TTTTTGCCTTAATTCTCGTGCGAGGTTAATTCTGCCCCACATAGCTCACTCACCCACCCCTTTCAGATTAAGCAA
TTTTTGCCTTAATTCTCGTGCGAGGTTAATTCTGCCCCCCTACCGGAGTTTTCAATCCAGTTAATAGGCGTGGAA
TGATGTGCCCCAACCAAACGACCGCCATGCACAACTTCCCTACCGGAGTTTTCAATCCAGTTAATAGGCGTGGAA

14

(7) 54595424 | 55206654 (7)

GCATTCCATCCTGGGATCACTAAACTCATAACTAAATTTTTAAAATTTGCATAACTAAAGTTCATTCTTTATGCT
GCATTCCATCCTGGGATCACTAAACTCATAACTAAATATGCATCCTTTTATCAGGGACTCACTCCCGTGATAGCT
GCATGTGAGGTGGTGGGGAAGAGAAAAAGCGGGTTTAACTCATCCTTTTATCAGGGACTCACTCCCGTGATAGCT

15

(7) 54659764 | 55123258 (7)

TGCCTTGTTAAAATCTCCTTTGTTACAGGAACTCTTAAAAACTTAAGGTGCCTCCAAGGAAAAATTATTA
TGCCTTGTTAAAATCTCCTTTGTTACAGGAACTGTAACATGAGTGGTATATGCATAATGGCATAGGTTAT
ACCAGCAAGATTTATGAACCATTTTCTGCTGTTGTATATAACATATCATATGCATAATGGCATAGGTTAT

16

(7) 54668755 | 54730348 (7)

ATTGGTGTCTAATTTATTGACCAGAAAAGAAACAAGGAAATTCTCTCAGCAGTTGAGTCTATTAAAGAGTCTATC
ATTGGTGTCTAATTTATTGACCAGAAAAGAAACAAGGACCTGGCCTCTGTGGAGATCAGGGTCCACTCAGGGCAG
TCTCTGGAACGCGACGGTGGAAGGCAGGATGCTGTCTGCCTGGCCTCTGTGGAGATCAGGGTCCACTCAGGGCAG

17

(7) 54671909 | 55208181 (7)

TCTTTGTCCCTGTTTCCTGGAATACAAATCTTAAAATCCTTGAAATCTTCAAAGTGCTATCTTTTCTATGCTAAT
TCTTTGTCCCTGTTTCCTGGAATACAAATCTTAAAATCGGTTGGCACACCTTGGAATAAAAGCTGCTTTCCCAGC
TGTAGGTACCAGGTTGTGACAATAACATGTGGCCTTTCGGTTGGCACACCTTGGAATAAAAGCTGCTTTCCCAGC

18

(7) 54697888 | 54701077 (7)

TCCCTAATGGTGTTAAACCTCTTTTGATATGCTTATTTTCCCATCTGTAAGTCTTCATAACATCTTCAG
TCCCTAATGGTGTTAAACCTCTTTTGATATGCTTATTTATCCTATAGTGGGAAATGAAGTGAGATCCCT
AATGAACTAATCAATAAATGGGGCTTGCACAATTATTTATCCTATAGTGGGAAATGAAGTGGGATCCCT

19

(7) 54701041 | 130190336 (7)

ATCCTATAGTGGGAAATGAAGTGGGATCCCTGCATCACATCGTATATAAATATCGTTCTCTGATTCGTTAAAATC
ATCCTATAGTGGGAAATGAAGTGAGATCCCTCAATTCCATCCCCTGATGGCTTCCTCTCCCACTTTTCCCATGAA
ACTAAGTTGGCTTAGACACCCTCTGCTGACTCAATTCCATCCCCTGATGGCTTCCTCTCCCACTTTTCCCATGAA

20

(7) 54713621 | 128958218 (8)

GGTTGTGAGAACAAGCCTCCCAGATGGATGGCTTTCTATACAAACCCACTCATAGCTCACACCTCTTGGCATGCCCAG
GGTTGTGAGAACAAGCCTCCCAGATGGATGGCTTTCTTCGCTGCGGGTTGGAAAAAAGAAAGAGTTAGTGTAACACAT
TTTGTTTCTCTGTAATAAAGGACTAAAGCTACCAGCCTCGCTGCGGGTTGGAAAAAAGAAAGAGTTAGTGTAACACAT

21

(7) 54738501 | 55259259 (7)

GGCTGTGTGAGTGTGTGTGTGTGTGTGTGTGCACATGCACATTCTCTAAGAACTGAGTTCTCTTTTGGTTAATTCTAG
GGCTGTGTGAGTGTGTGTGTGTGTGTGTGTGCACATGCAGAAAGGCGCATGAAAGAAGATGTAACCCGCTGCTGACCA
ATGGCTGGCGAACGTTAGTGACTACTGATCCAAAGAATGGAAAGGCGCATGAAAGAAGATGTAACCCGCTGCTGACCA

22

(7) 54748976 | 54758053 (7)

CACTGATGTCGTAGGGAAGACCAGAACCTGATTGGAGTGGATTTTGGAGGGAATGGGAGGAGAGAAATTGGAAACAA
CACTGATGTCGTAGGGAAGACCAGAACCTGATTGGAGTGGACCAGTCGTGCTGACTCGGATTTCGAACATTTCTCCT
CTGTTCTGGACACAGCAACGACGGGAGAATTCCCATCCTGACCAGTCGTGCTGACTCGGATTTCGAACATTTCTCCT

23

(7) 54748977 | 55257873 (7)

ACTGATGTCGTAGGGAAGACCAGAACCTGATTGGAGTGGATTTTGGAGGGAATGGGAGGAGAGAAATTGGAAACAACA
ACTGATGTCGTAGGGAAGACCAGAACCTGATTGGAGTGGGAAGTGTTGTCCAGAGAAGAACCTGCTCATTTCCTCTTA
CTTTCCACCCACCATCCTGACATAATACTTCCTAATCTGGAAGTGTTGTCCAGAGAAGAACCTGCTCATTTCCTCTTA

24

(7) 54749077 I 55202557 (7)
CAAAAAAAGAAAGATTAATTGATCTGTCTATGTACATAAATGGCAAGCCAAAAATTTCAGTTAAGACTGAGAAATAA
CAAAAAAAGAAAGATTAATTGATCTGTCTATGTACATAAATGATCTCAGTACTGCTCACAACTGCCCTGTGAAATTC
TTTAGAGAGATCTATGATTTCTGAGGCCCTTTCATGTCCATGATCTCAGTACTGCTCACAACTGCCCTGTGAAATTC

25
(7) 54749087 I 55262345 (7)
TCAAAGTCAAAAAAAGAAAGATTAATTGATCTGTCTATGTACATAAATGGCAAGCCAAAAATTTCAGTTAAGACTGAG
TCAAAGTCAAAAAAAGAAAGATTAATTGATCTGTCTATGACGGGAGGGGGACCTGGTGGACGGGAGAGTTGCCAGGGC
GGCATTGCGCTGGCACACACAGGCTATGCCTGGAGCGTGACGGGAGGGGGACCTGGTGGACGGGAGAGTTGCCAGGGC

26
(7) 54749352 I 55259872 (7)
ACCCCAAGTTACATCCCTTCTTTATGTCGTCTTTCTTAATTGAGACAGTGCTGTTTATTGAAGGTGGCATTCTGTTTT
ACCCCAAGTTACATCCCTTCTTTATGTCGTCTTTCTTACTGGGAAAGGTCACAGCTGCCTTGGTGGTCCACTGCTGTC
CTCCCTGCAGGATATATAAGTCCCCTTCAATAGCGCAATTGGGAAAGGTCACAGCTGCCTTGGTGGTCCACTGCTGTC

27
(7) 54880905 I 54986016 (7)
AACATCATAAGTGCTCAATATAGAATCCTATTTTTATTACAATTATCATTTTTCCATTTGTTTTCATCTCACCGTCAT
AACATCATAAGTGCTCAATATAGAATCCTGTATTCATATGCATAATGGCATATGAATTACAAGACATACTACAACATA
ACTATTTGTAGCAATTCCTTTTACCTGGGACATTATGTCCTGCCATCAAGAAAAAGTTACAAGACATACTACAACATA

28
(7) 54882728 I 127757114 (8)
TTAGTTCCCATACATGAGTGAGAACATACAATGTTTGGTTTTCCATTCCTGAGTTGCTTCACTTAGAGTAATAGTCTC
TTAGTTCCCATACATGAGTGAGAACATACAATGTTTGGTATACTGGAGGTTATATCGTGAAACCCAGGTGGGGGCTTC
ACTCTTCCTGACCCCAGCCCTGGCCAAGGGAGTTTGTTCCTACTGGAGGTTATATCGTGAAACCCAGGTGGGGGCTTC

29
(7) 54928591 I 55278959 (7)
GTGTGCCTCACTCACTGAGCTCCCCTCTTCTTTTCCCACATGCAATCTGTCTGCTGGAGGTCACTGAACCCTCCCTTC
GTGTGCCTCACTCACTGAGCTCCCCTCTTCTTTTCCCACACCAAATCAGGTCATAGGATTCCTTTTTTTTTAAAATAA
TTCCAAGAGATTGTATTGTACAGCAAGATTATTTTTGTGGCCAAATCAGGTCATAGGATTCCTTTTTTTTTAAAGATA

30
(7) 54928835 I 55268502 (7)
TTTTAGAAACCTTTAATCTTTTTCTTTTCAAGTATGGCCACTAGGAGTGATTTTGGCCCATTTAATAAAAAATGAGGA
TTTTAGAAACCTTTAATCTTTTTCTTTTCAAGTATGGCCAGGAATACCAAAACCAAGCTCACAGGATTGTCTCAAAGA
CACCCAGGCAGCCGATCCACCTATCTCCTTCCATAACACAGGAATACCAAAACCAAGCTCACAGGATTGTCTCAAAGA

31
(7) 55064954 I 55280092 (7)
CCCTGCCCTGGAAGCTCAATGGTGCCAGCACTGAGCTACATCCACTGGCATCTTTCTACTTGCCCCCACTGACTTCCT
CCCTGCCCTGGAAGCTCAATGGTGCCAGCACTGAGCTACACAGAGTGTACTTCACACATCTAGATGGCACAGCCCAGC
AAATGCATCATTAGGAGATGTCATCACCGTGCGAGCATCACAGAGTGTACTTCACACATCTAGATGGCACAGCCCAGC

32

(7) 55065340 | 55262340 (7)

AGCTGCACCTGCCACTCGCTCTAGGCTTTTAGAAATGGAGCCTGTAAATTCTCTCTCTCTCTCTCTTTTTGGCTTAA

AGCTGCACCTGCCACTCGCTCTAGGCTTTTAGAAATGGAGCCCGTCACGCTCCAGGCATAGCCTGTGTGTGCCAGCG

CATCTGGCCCTGGCAACTCTCCCGTCCACCAGGTCCCCCTCCCGTCACGCTCCAGGCATAGCCTGTGTGTGCCAGCG

33

(7) 55066261 | 55258204 (7)

CTTTAGTATTTGGACTATTCCTAAATAATAGCTTTAATAGTATACTCAGTGGGCCCAAGGATACATTTTTGTGGATCC

TCTGACATGCCAATTCAACAAATCATTTTCACATAATATTCATGCAAAAAAAAAACAATTTGCCAGAAAACTTGGGAA

TCTGACATGCCAATTCAACAAATCATTTTCACATAATATTCATGCAAAAAAAAAACAATTTGCCAGAAAACTTGGGAA

34

(7) 55123280 I 54720579 (7)

ATATGCATAATGGCATAGGTTATTGTTTTCTTCAAAATATATGAGATGTGAGTCCTTCTACG

ATATGCATAATGGCATAGGTTATATAGTAGCTCTTACTATCTTTCTCCAGTGCCTTTGCACT

ATACAATAAAATCTGAAAAACAGATAGTAGCTCTTACTATCTTTCTCCAGTGCCTTTGCACT

35

(7) 55125526 | 55258369 (7)

GTGAGGTTAGCGGAGGCAGAGGCCTCTCAGGCCTGAAGATAGCCTCTGTTTTCAGGGAAATACTAGACTGTGAGATCT

GTGAGGTTAGCGGAGGCAGAGGCCTCTCAGGCCTGAAGAAATGCTTGCTGCGGTGCAGCAAACAGAAACAAACAGGCA

ATGTTCATGTACTTTCTAGTACAGAGTGGGCACGACAGAAATGCTTGCTGCGGTGCAGCAAACAGAAACAAACAGGCA

36

(7) 55206720 | 54701168 (7)

GCTAACCCATTCTTACATGAATGGCATTAATCCATTCCTTAGGGCACAGCTCTCATGACCTAATTATAATACCTC

GCTAACCCATTCTTACATGAATGGCATTAATCCATATGTTTCTGTTTCTAAGGTGTATTTACTTCATTGGTCAGT

CCACCTTGAGATATGCCAGGTGTAACCATATATGTCTGTTTCTGTTTCTAAGGTGTATTTACTTCATTGGTCAGT

37

(7) 55241284 | 55269336 (7)

CGGTAGGGAAGTTGTGCATGGCGGTCGTTTGGTTGGGGCACATCACACGCGCTTATGCTTATGAGCCGCTGGGTTGC

CGGTAGGGAAGTTGTGCATGGCGGTCGTTTGGTTGGGGATATTGTGAAGGTGTTTAATTTTTGTCTTAATCGTTAGT

ACTTTTCCTGAATGCAGGTGGTGTGCCTCATGGAGGGTATATTGTGAAGGTGTTTAATTTTTGTCTTAATCGTTAGT

38

(7) 55262427 I 55268831 (7)

CTCCATGCACAAGTGGAAGCCCTCTCAAAGTCAGTGGCTTAGTGCCTTGATGTGGTCACACCCATTCTCAGGAAGTC

CTCCATGCACAAGTGGAAGCCCTCTCAAAGTCAGTGGCTTACCACTTCTTGGCTATAGGAGCAGGGGTCTATTGCCA

GAGATTTTGAGGCTATTAGAATGGCCCGTAGAGATGCTATTCCACTTCTTGGCTATAGGAGCAGGGGTCTATTGCCA

39

(7) 55262553 | 55262169 (7)

GCAGATTATAATCACTGGCCTAGGCAGCCCACTGGAACTACCAGACCATGAGCCTGAATTTTTTGTTTAAAAATCATA

GCAGATTATAATCACTGGCCTAGGCAGCCCACTGGAACTATCGGCCTCCCAAAGTGCATATTTTTTAACTTTATCAGA

CAGGCTGGTCTCGAACTCCTGACCTCATGATCTGCCCGCCTCGGCCTCCCAAAGTGCATATTTTTTAACTTTATCAGA

40

(7) 55315470 | 47338022 (7)

AGTTCTGTGCTGAAAAATGTAGTTTCTTTTCATGGTGTGACCTTTTTATGAGTCCTAAGTATAGACTGGCCAT

AGTTCTGTGCTGAAAAATGTAGTTTCTTTTCATGGTGGGTTAGGGTAATAGTAACCTCAACTCGATACCGCCC

CATGGCCAAAATCTGGCATTTCCAAGAAGAGGGAGTGGGTTAGGGTAATAGTAACCTCAACTCGATACTGCCC

41

(8) 127825883 |128579780 (8)

CTGTGTATGACAAAATGCAGTAAACCAAGTAAAAAACCTGGAAAGGATATTTGCCATGTATATTATGAAGAAACTGGT

CTGTGTATGACAAAATGCAGTAAACCAAGTAAAAAACCTGTACACGTACAGCAGGACTGCACACTTATATAGAGTCTG

TCCCCTCTTAGGCAGCCGTGGAAGCTGCAGTTAGGGCATTTCAAGGGACAGCAGGACTGCACACTTATATAGAGTCTG

42

(8) 127878972|132198438 (8)

CACGAAAAACTTCGAAAAGCCCTAAATATGCCAGAGAATACAAATTCAAAAGAAAATATGGAATCCAGGGAGATGGGG

CACGAAAAACTTCGAAAAGCCCTAAATATGCCAGAGAATATTTAATATCCCAAAGCTGCACAGGGCAATGGGTCTGAA

TTTGAGCTGAGGATGGAGAAGGAACAGCCAAGATAAGGGGAGCAGTATCCCAAAGCTGCACAGGGCAATGGGTCTGAA

43

(8) 128093657 | 128687565 (8)

TACTAGCCTTTTCCACAATGGGGAAGAAAAGTCAATTAAATCACCCCTGGACACAATTTACATGTCTATAGACAAGAA

TACTAGCCTTTTCCACAATGGGGAAGAAAAGTCAATTAAATCCTTAGCTGTGAAGAACTTACCAGGGCTGCCTATTAT

CTGAATTGAATTGGCTTATAAGAAAGACATAATATTTAAATCCTTAGCTGTGAAGAACTTACCAGGGCTGCCTATTAT

44

(8) 128433071 | 129473548 (8)

TCTCAGTGATTTTAAGTAAAGATACATTAATTTTTTACTTACGTCAGTCCAAAATAGGGTTCCTGAGGAGCAAGTGGC

TCTCAGTGATTTTAAGTAAAGATACATTAATTTTTTACTTCCAATGTATCTTTTTCAATCACAGGCCTGCAAATTCCC

CCTTCTCTCTCTATCACCAACCAATTCTCCCCAGAAATGACCAATGTATCTTTTTCAATCACAGGCCTGCAAATTCCC

45

(8) 128460114 | 131055616 (8)

GGCAAAGGTACAAAGTTGTGGAGATGGCAGATTGCTCTCAAGGTGCCCACCTGATGATTCATGCTAGGAATTTATGCA

GGCAAAGGTACAAAGTTGTGGAGATGGCAGATTGCTCTCTCCTGACCTCAGGTGACCTGCCTGCCTGGGCCTCTCCAA

AGACGGGGTTTTGCCATGTTGACCAGGCTGGTCTCAAACTCCTGACCTCAGGTGATCTGCCTGCCTGGGCCTCTCCAA

46

(8) 128503400 |129374147 (8)

GAAATGAGAAAAATAAATTATTTGAGCTCATTTATATGTCATAGGGGTACGTAACTTCACAAAAATCCCACAGCGTTT

GAAATGAGAAAAATAAATTATTTGAGCTCATTTATATGTCTCCTCCAGAGGCCTGGCAGTGTTCTCTTTAGGCTTCAA

ACACCAGAGGGGAGAAATGCTGGAAATAGATGCAAACTCTACCTCCAGAGGCCTGGCAGTGTTCTCTTTAGGCTTCAA

47

(8) 128579178|131739197 (8)

GATCCTCAAGGTCACCCAACTAGCAGGTGGGAAAGCCAAGATTCAAGTTGAGATCTTTCTAACTCCAAA

GATCCTCAAGGTCACCCAACTAGCAGGTGTTTCTAGAAGATAACATTGGAAAAACTCCTTTAGAAATTG

AAAACAGAAATGAACCTGAAATCATAAAATTTCTAGAAGATAACATTGGAAAAACTCCTTTAGAAATTG

48

(8) 128984176 | 129545398 (8)

ATGCTGGTCTTCGGACAGTCCGGCCAAGTGAGACTGTCAGGCAGATGACTGTGGTGAAGATGGCCCAGCAAGCCCCTC

ATGCTGGTCTTCGGACAGTCCGGCCAAGTGAGACTGTCAAATTAACTGCCAAATAAAAGGGGAACTCTTCAGCAAACT

CATCCAAATAACACACACTGTTTGCTGCTACTGGTTCTTTATTAACTGCCAAATAAAAGGGGAACTCTTCAGCAAACT

49

(8) 129139899 | 131199290 (8)

CTCAGAAAACATAGTGTATGGATCTGGCCAACAGAGTCCTTCAGTCTCCTAAAAGTGTATGCAAAACATTGTGTATGA

CTCAGAAAACATAGTGTATGGATCTGGCCAACAGAGTCCTCCACTTTAGGTCAGTGTACCCAACCTCTTAGCCCTAAT

AAGTACCTCTCCCTTCAGACAAATAGCACAGGACATGCAACCACTTTAGGTCAGTGTACCCAACCTCTTAGCCCTAAT

50

(8) 129465157 | 129471271 (8)

CACCTGCTTCACAATGAATTCCTTCTTTCTAGCTATCCAGCTTGTGTTTTTTTTTTTTTTTTTTTTTTTTTTTTTTTTTA

CACCTGCTTCACAATGAATTCCTTCTTTCTAGCTATCCAGCTTGTGTTTTTAAACCTTATTTCATCACAGCAAAAACT

GAGCTGTTCCTATTCGGCCATCTTGGCTCCTCCCTCCCAGCTTGTGTTTTTAAACCTTATTTCATCACAGCAAAAACT

51

(8) 129546684 | 132578151 (8)

CATGAACTATTGACAGGAACATCATGTATAGAAAAGGCAGGTCTTGATACATATTTTTGTGTGTGCATCAAATGCATG

CATGAACTATTGACAGGAACATCATGTATAGAAAAGGCCATTTAACCATGGTAAATACATGGTTAAACATAGATGAAA

TACATCAAATGAGGTGTATTTTCTGCAAGATTAAAGAGTCAAGGAAAATGGTAAATACATGGTTAAACATAGATGAAA

52

(8) 129735150 | 132827500 (8)

GAAATAATCATAGCATATTACATTTTTAGATTGtagattgtattcacatacattatgtcatataagcttt

GAAATAATCATAGCATATTACATTTTTAGATTGAAGTGAATCAGAATTCATCTTTCAAATTGCAAAAAGT

tctacagaacatttctatatttagagccatatctgtgtttTCAGAATTCATCTTTCAAATTGCAAAAAGT

53

(7) 130190400 | 128957699 (7)

CTCCCACTTTTCCCATGAAAATTTGTTTGTATTTTAAAAACCGGGCTTTTACAAAGATTTAGATGTTGCTGCCAC

CTCCCACTTTTCCCATGAAAATTTGTTTGTATTTTAAACCTATCAAATGTGGGTGACCAGATGGGGGAAAACAGG

CATCACCCCCATGAGACTGGCAAAAAGCAAAATTCTGACCTATCAAATGTGGGTGACCAGATGGGGGAAAACAGG

54

(8) 130612941 I 132581669 (8)

AGGCTGGGTGGCAAAGTAAGACACTATCTCAAAAAAAAAAGTATCAAAAATAATATTATGAAAATGGCCATACTGCCT

AGGCTGGGTGGCAAAGTAAGACACTATCTCAAAAAAAAAAGTTTGTTGGCCATTTGTATTATCTTCTTTTGGAGAATT

GCATTTCCCTGATCATTAGTGATTCTGAGCATTTTTTAATGTTTGTTGGCCATTTGTATTATCTTCTTTTGAGAATTG

55

(8) 130923423 | 128579150 (8)

ATGTGAAATATATCATGCAGTACAGTGTCTAAAACATAGGaagtacttagaaaaaggcagctgagtagaaagggac

ATGTGAAATATATCATGCAGTACAGTGTCTAAAACATAGGtaagcaaGATCCTCAAGGTCACCCAACTAGCAGGTG

tcctagtttctagatgaggaaactgaagctcggaggctcagagatgcGATCCTCAAGGTCACCCAACTAGCAGGTG

56

(8) 131104145 | 131928335 (8)

ATGTGTGTGCATTTTGGCACACTTTCTGAATGTGCTAACAGGCCACTGCCCAGTGGAGCCCCACACGCTGCCTGGCCC
ATGTGTGTGCATTTTGGCACACTTTCTGAATGTGCTAACTCAGTTCTCTCACTTGTAAACTGGCTCTGGTAGTGATAC
CAATAACTGTGTGACGTAAAGCAATATACCTCTCGTGACTCAGTTCTCTCACTTGTAAACTGGCTCTGGTAGTGATAC

57

(8) 131850749 |131852739 (8)

TAAGAAGTTCATTCTCCAGAGCTCATTTTCTGCTCCAGAGAAACTCTAAGGCCCTCCAGTTTGACAAAGGCCAAAGAA
TAAGAAGTTCATTCTCCAGAGCTCATTTTCTGCTCCAGAAACAAGAGTTTTCAGAGGAGCCTTTTAAATGACAACAAA
AGTGAAGAAACATGTGCCCGAAGAATAAAGAGCTCTATATACAAGAGTTTTCAGAGGAGCCTTTTAAATGACAACAAA

58

(8) 131862826 |131868801(8)

TTTGCTGCCTGAAATAGAGATGGAAAAATTAGAGCTGCCAGTGTAGAACATGAATGGCTACACAGAGTTGAGAACAAA
TTTGCTGCCTGAAATAGAGATGGAAAAATTAGAGCTGCCAAGGACCCTAGTCCTCATTGTCTCTTAGGTAGTCAAGAA
ACCAACACTCACAGCTCGCCACCCCGAGGTGCCTTCGGTCAGGACCCTAGTCCTCATTGTCTCTTAGGTAGTCAAGAA

59

(8) 132071238 |132148229 (8)

CTTCAGGGGCCAGCCCAGAGGCTCAGTCTTCACAATACTGGATCCTTCTGTTCTATCTGTGGTTGCTATGCTCTTCTA
CTTCAGGGGCCAGCCCAGAGGCTCAGTCTTCACAATACTGAAGAGTCAGATCTGCAACTGGGACTTCAAAACAATAAA
GGGTGGTACAGAATGTCTGCTTTTCCCAAGATCCTCAGCCAAGAGTCAGATCTGCAACTGGGACTTCAAAACAATAAA

60

(8) 132277696 | 132280638 (8)

AAAACACCAAAATCAATGGCAACAAAAGCCAAAATTGGCAAATAGGATCTAATTAAACTAAAGAGCTTCTGCACAGCA
AAAACACCAAAATCAATGGCAACAAAAGCCATCAGAGTGAACAGGCAACCTACAAAATGGGAGAAAATTTTCACAACC
AAGAGCTTCTGCACAGCAAAAGAAACCACCATCAGAGTGAACAGGCAACCTACAAAATGGGAGAAAATTTTCACAACC

61

(3) 111274086 |128533830 (8)

TCTTCATATGTGAGTAAATAAAAACTATACATACATTTATTTTTCTTTTCTTTTCTCTTAATTTTGTTAAAGAACAT
TCTTCATATGTGAGTAAATAAAAACTATACATACAAAAAAAATAAAAAAAATAAATGGTAAGCTATATAATATTGT
TTTATGGATAATATTGTAAAACTTAATGGAAAGACATTAAAGGAAATCTAAATAAATGGTAAGCTATATAATATTGT

62

(8) 130152661 | 114484890 (8)

TGAGGTGGAGCTAATTAATATTCACTTCAATGTAtcttataaagttatttttgaagatcaagagaattaa
TGAGGTGGAGCTAATTAATATTCACTTCAATGTAAAAATTGTAAAAATGTAAAAATatcaagagaattaa
aggaagtgattccatccatttggaagaaggcaattAAATTGTAAAAATGTAAAAATggatactattttcc

63

(8) 114484910 | 127698232 (8)

gaagaaggcaattAAATTGTAAAAATGTAAAAATggatactattttccagcttacagtaactatgcaacaaag
gaagaaggcaattAAATTGTAAAAATGTAAAAATTACCAAGAACCAACACACATGTATACTTGCTCTATGAAA
taaattctggatagtttgttatatagcaatagATTACCAAGAACCAACACACATGTATACTTGCTCTATGAAA

66

(7) 54427305 | 54918735 (7)

TGGTTGTTTCCCTTCTAATGGTCACCTACCAAAGGGGCAGAATAGAGAAAACCCATCTTATTCCTATTACCACAAAT
TGGTTGTTTCCCTTCTAATGGTCACCTACCAAAGGGGTAGAACAATGGAACAGAACAGAGGCCGCAGGAATAACGTC
ACCAAAGCAGCATGGTATTGGTACCAAAACAGATATATAGAACAATGGAACAGAACAGAGGCCGCAGGAATAACGTC

67

(7) 54438342 | 55044062 (7)

TTCTTTCCTATTAATATCTAGTACACCTAGTTACATTTTTTAAAAGTCATTTCCCATTACACTGAAGGGTCACTTTG
TTCTTTCCTATTAATATCTAGTACACCTAGTTACAATTACATTAATAAACCACATATTTACAGCCAACTGATTCTGAC
GAAATGACACAGAGATCAATGGAACAGAATAGGGAACCCAGAAATAAAACCACATATTTACAGCCAACTGATTCTGAC

68

(7) 54438551 |54490561 (7)

ATTTTTCAAACAATGCACAAAAAGCACTTAACAGAGATGAAAATCTTGACAAATTATATTAGACTAAATTTAAGAGC
ATTTTTCAAACAATGCACAAAAAGCACTTAACAGAGATGATAATTTTGTTGAATGGAAAATCACTTATCAGAAAATC
TGAGAAGGCTTCACAAAATAATGTTTCTGGATATGGAAATAAATTTTGTTGAATGGAAAATCACTTATCAGAAAATC

69

(7) 54439588 I 55292449 (7)

CAGAGGCCACATTGCACCTCCTCCCAGCCAGGATTTATCCACACAGCTAGCCTTTGTTACAACCATCTGCTCTCCTA
CAGAGGCCACATTGCACCTCCTCCCAGCCAGGATTTATCCAACAGGGCAGCTCCTGTAGCGACTTTCTCCTTGTTTT
TGTCTTTTACTGAACAGGCAATCCTTCCACTTAGATCCAGAACAGGGCAGCTCCTGTAGCGACTTTCTCCTTGTTTT

70

(7) 54451802 | 128484872 (8)

AAAATGTATTACCTTCACTCTGGCCTAAAGCTAGTTGTcttccacagccttttacttcttcctgagttttcacca
AAAATGTATTACCTTCACTCTGGCCTAAAGCTAGTTGTACCTTAAATACCAAGTGTTGTTCAGCAAACATTCATT
ctagaacacatattgcctgccactgataatttaGTTGTTCAGCAAACATTCATTTGTTGAGTTGCACGCCAGACA

71

(7) 54488433 | 54966885 (7)

TATGCATATTTAAAAGACTAAATAGAATATATATAGATTTTTTTGAGACAGAGTCTCACTTTGTTGCCCAGGCTGGA
TATGCATATTTAAAAGACTAAATAGAATATATATATTCTATTTATATATAAATAGAAAAACAAGGCTCAGCAGAATC
TAGACTGAATAAAGCCAAGCCATGAGTCACATGTCCACACAACCAGTTACCCGAGAAAAACAAGGCTCAGCAGAATC

72

(7) 54496942 | 130152776 (8)

TGGCATTCCTCTTTAATTCTGTCCATTATTTGGAAACCTCTGTCATCAACATTCAGCTTCACTGAACAGGCTGAAAG
TGGCATTCCTCTTTAATTCTGTCCATTATTTGGAAACCTCGTAAAGTCCTGTCTCTGCTACCTGTTTGCTTCATTCC
ATAACTGAACAATAAAGAATTTGGAATTGGAAGACTTGCTGTAAAGTCCTGTCTCTGCTACCTGTTTGCTTCATTCC

73

(7) 54559825 | 55280053 (7)

CTGTTCTGGGGATAAAGGTGTTCTCGCAGGTGTGCCTTCCTGACACCTTAACCTCCTATTTTTTAGTCTCTAAAACC
CTGTTCTGGGGATAAAGGTGTTCTCGCAGGTGTGCCTTCCTCAGAACATGCTCCTGTGGTAAGGAAGCCTGACTGCA
GATGCTCGCACGGTGATGACATCTCCTAATGATGCATTTCTCAGAACATGCTCCTGTGGTAAGGAAGCCTGACTGCA

74

(7) 54657308 | 55292468 (7)

TTCAGATTCCTCACTTATAATGACAGTGCTACATTAACAACAGCAACAACACATATGTACAGTATACACACATACAG
TTCAGATTCCTCACTTATAATGACAGTGCTACATTAACACTGAGCTACAGGAGCTGCCCTGTTCTGGATCTAAGATG
TTTGATCACTGAGAAGTTAATTTCACAAAACAAGGAGAAAGTCGCTACAGGAGCTGCCCTGTTCTGGATCTAAGTGG

75

(7) 54670874 | 54439660 (7)

GATTTGGAATTGTGTGCCCTAGAATCAAGGTTATTTGCAAATTTATTTTGCCAAAATCAGAAACCAGGTTTGAGAGAC
GATTTGGAATTGTGTGCCCTAGAATCAAGGTTATTTGCAAATCTTCAGATGGAAAATCAGGGCATTTCACTTCAGAGG
TCTCATTGAATTCCCACGTAGGAAACACACCCATGTAACCATCTTCAGATGGAAAATCAGGGCATTTCACTTCAGAGG

76

(7) 54765765 | 128648771 (8)

GATGGGGAGGGAGGCAGCAACGGTGACAGCTCCTCCCAGGCTGCGTGTCCCCTGTGTTCTGGTGAGAGCCAGAGTGTT
GATGGGGAGGGAGGCAGCAACGGTGACAGCTCCTCCCAGGAGCTAAACCCTCAGAGCATCCCCCAGCTAGTAGAAGAG
ACGATGCTAAGCATTGGGAGTAAATGAAGAATAAAATGCAGACTAAACCCTCAGAGCATCCCCCAGCTAGTAGAAGAG

77

(7) 54957228 | 54973297 (7)

TTATACGTGAACCATCACTGCACATGCTAGATACACAATAAAAGTTAGTGCCATTCTTTCTCCTCTCTTCTTATATG
TTATACGTGAACCATCACTGCACATGCTAGATACACTACATATATAGACATCTCCAAAGGCTTGTTTTTGTTTAACT
TAAAGTCTTAAAAGTCACCAAACAGAAAAGCTGAGAAACCCTACTAGACATCTCCAAAGGCTTGTTTTTGTTTAACT

78

(7) 54960833 | 54957193 (7)

TCTCTGCTGTATAAGCTCTACTTTGAAAACATGGGTCTTTTTTTTTTCTTTCTTTAGCTTTAAGAGATTACATAGTA
TCTCTGCTGTATAAGCTCTACTTTGAAAACATGGGTCTTGTTTATACGTGAACCATCACTGCACATGCTAGATACAC
CCTCATTGCCCCTCCTCTGCCTCAGCCCAGGACACAATGTATTATACGTGAACCATCACTGCACATGCTAGATACAC

79

(7) 55011056 | 128016981 (8)

TGGTGCGTGCCTGCAGTCTCAGCTCCCAGCTACTTGGGAGGCTGAGATGGGAGGATCACTTGAGCCCAGGATAGGGA
TGGTGCGTGCCTGCAGTCTCAGCTCCCAGCTACACAGACAGGATGATCTGATAGCTCATCCCTAAACTAAAGTGTTT
CTTCCCTGAATGCCCATGTAGGAGAATAAATGATATTAGGAGCTGATCTGATAGCTCATCCCTAAACTAAAGTGTTT

80

(7) 55207522 | 55280274 (7)

ATGACGTAGAATTTAAGCATACTTACTTGTTTAAACAAATTGTCCAGTTGCTTCCCAAAATGTTTTGTGAATTAAGA
ATGACGTAGAATTTAAGCATACTTACTTGTTTAAACAAATTGTCGTTGTGTCTTTTTAACATTTAGCTATGTTTATA
AACGATGTTCACATGGTCCCATATGATTATAATACCACGTTTTCACTGTGTCTTTTTAACATTTAGCTATGTTTATA

81

(7) 55216848 | 128458972 (8)

GTAGCCATTAACATATTTTACTTGAAAGTTTTCTTTTATGATGTCAGATTTTGGCTTTTCTTCAACTTGATATTACAA
GTAGCCATTAACATATTTTACTTGAAAGTTTTCTTTTATGAATGTGACTCTGTCTCTAAATAAATAAATAAGAAAAAG
GTGAGGCGAGATCGCCACTGCACTCCAGCCTGGGTGACAGAATGTGACTCTGTCTCTAAATAAATAAATAAGAAAAAG

82

(7) 55262174 | 55207419 (7)

TGAAAAGTCTGATAAAGTTAAAAAATATGCACTTTGGGAGGCCGAGGCGGGCAGATCATGAGGTCAGGAGTTCGAGA
TGAAAAGTCTCATAAAGTTAAAAAATATGCACTTTGGGAGTGAGCAACATTCTGCATAAGGTCTGTTTCCTCAGAAT
TAAGTTTCTTTGCAATTTTTTCCATTATTAACAAAATTAATGAGCAACATTCTGCATAAGGTCTGTTTCCTCAGAAT

83

(7) 55279982 I 55125302 (7)

CTGCAGTCTGAGCTCGGACTCCCTAGAACCCTCCCCTGCCACACCGTGGCTATTCCCGATGTGCAGGTAGTGGCTTC
CTGCAGTCTGAGCTCGGACTCCCTAGAACCCTCCCCTGCACTTTTGTGCAAAATGCAGTTTTACCAGCCTCTTTCCT
TTCATTAGAACATATTATTTGACTTCATGTTGAATCAACACTTTTGTGCAAAATGCAGTTTTACCAGCCTCTTTCCT

84

(7) 55286758 | 55289062 (7)

TGTATTGCTTACAGGAACCAGCAACAGAAAGAGTTGCAATGTGCTTTTATTTTTTTTTTTTTTTTTAATTTATTTTT
TGTATTGCTTACAGGAACCAGCAACAGAAAGAGTTGCAATGTGCTTTTAAAAGGGAGTTGAAGCAGCAGGGTGTTGG
GGGAGAGGGAGAGGGAGAGGCTAATTGGCAACTCTGCAATGTGCTTTTAAAAGGGAGTTGAAGCAGCAGGGTGTTGG

85

(7) 55292438 I 128233714 (8)

GCTACAGGAGCTGCCCTGTTCTGGATCTAAGTGGAAGGATTGCCTGTTCAGTAAAAGACAGGTGCAGGCAGG
GCTACAGGAGCTGCCCTGTTCTGGATCTAAGATGCCATTTAATAAATGATGGTTTTCTCTATCCTGCATCTT
GCTACAGGAGCTGCCCTGTTCTGGATCTAAGATGCCATTTAATAAATGATGGTTTTCTCTATCCTGCATCTT

86

(8) 132022399 | 55301671 (7)

AGTATGGGATTCCAAAATCCATTTCATGTCAATATgtccaattctgaacctcacaatatatggactttct
AGTATGGGATTCCAAAATCCATTTCATGTCAATATACGATGATGAAAGCTGGCACAGTCAGGGCGGTGCC
tcagctgaggggcagcagcaaccttgcaagctggTACGATGATGAAAGCTGGCACAGTCAGGGCGGTGCC

87

(7) 55306136 | 131240408 (8)

TAGACGGGGGATGCTGAgagagaagccggtttgctgggaggtgcgagcccagagcacacgctccacctagactgc
TAGACGGGGGATGCTGAttggaagctgtatatacctgtaatgtatggtatttgtTTGTTAAGAAATACTCAGTTA
aaaatcatctctgtttcactggtagaaaaaccaaggtcctgagaaacgaaggacTTGTTAAGAAATACTCAGTTA

88

(8) 128016842 |128458024 (8)

TATAATTCATAAGGAAAAAGAAAGAAAGAGAGAGAAGAGGAAAAAAAGGAAGGAAGAAATCCTGCCTTCAAGGACTT
TATAATTCATAAGGAAAAAGAAAGAAAGAGAGAGAAGAGGGATGAGGCTGCCCAGAGTCTTGTGGGGACCAACCCAT
TGAAAGCTGCAGCATAAGCCGCGCCTAGCAAAGCCACGGGGATGAGGCTGCCCAGAGTCTTGTGGGGACCAACCCAT

89

(8) 128310905 | 130393312 (8)

GGGTATTTACCCCAGACAATGATGCTGCTTCACTTACGCCAAACAAAAAAAGCACCGAATGTCCATAT
GGGTATTTACCCCAGACAATGATGCTGCTTCAATTTAAAATCACCAGCTGCATTAGCCCCAAACAAGA
ATTTATGAAATGGTAAACAAGCATTGGCTTCAATTTAAAATCACCAGCTGCATTAGCCCCAAACAAGA

90

(8) 128314386 | 129289585 (8)

TCGTGGAAGAGGTGCAGCCTTCGCCAGACCAGGTGCCACCCACTTTGTGATTAATTGATGTACCAGCTCCCCCTCTA
TCGTGGAAGAGGTGCAGCCTTCGCCAGACCAGGTGCCACCCTGTCCCTCTGAGTGGCAGCTGCAAGTTAGTCCGGTC
TTAGAGGAAAAGCTCACAGTGTGGATCTCTACATACTGCTCTGTCCCTCTGAGTGGCAGCTGCAAGTTATTCCGGTC

91

(8) 128401487 | 129032908 (8)

ATCACAACCGAGTAAAGTAACAGACTTTCATAACTCCAGCCTTCATAATTCAGTAGCTGCATATTCAT
ATCACAACCGAGTAAAGTAACAGACTTTCATAACTCATGAAGCTGAATTCAAGGTGACAGCGGACTGG
GTGGGTTGGCTCAGCTGGTTTCCCTGCTCCTGGTTCATGAAGCTGAATTCAAGGTGACAGCGGACTGG

92

(8) 128620473 | 130361150 (8)

GGTCATATAGTCAAGTGAGGCCACTTGAAGATGGAGGATGGAGCTGAGGAAGGCCACTTGAAGATGGAGGATGGAGC
GGTCATATAGTCAAGTGTCTGCCAGACACTTTGACTTTGAGAGTTAATATGTCATGTAGACTGCATATTTGTTCAGT
TGACCGTACGATGTACTGTGGTAAAATTTATGTTTCTGGGTCGATAAAAATTTTATAACACTGCATATTTGTTCAGT

93

(8) 128656514 | 131883074 (8)

TGACCCCTGGTTTAGAGCATTGAACTCTGAAATTGAAgagggctaggttagaaattcgaaatccagctac
TGACCCCTGGTTTAGAGCATTGAACTCTGAAATTGAAAGGACTTTGCTTTTAGATTTGTCACTATTTTAT
ttcagatggcaaattgggtggagacaaagtacggagacgtACTTTGCTTTTAGATTTGTCACTATTTTAT

94

(8) 128728355 | 130683305 (8)

GGCTGAGGTAGGAGGATTGCTTGAGCCTGGCAAGTTCAAGACTGCAGTGAGTCGTGATCACACCACTGCACTCCA
GGCTGAGGTAGGAGGATTGCTTGAGCCTGGCAAGTTCAGTTTTATTCTTAGATATTTACCTAAGAAAAATGAAAA
TATCATATAATCTGGCAGTTTCATCATATAATCTAGCAGTTTTATTCTTAGATATTTACCTAAGAAAAATGAAAA

95

(8) 128800883 | 128890626 (8)

ATCCCAACACTTCGTAAGATTGAGGCAGGTGGATCTCCTGAGGTTAGGAGTGTAAGACCAGCCTGGCTAACATGGTG
ATCCCAACACTTCGTAAGAATGAGGTGGCACCCAACTTTTCACTGTACTTCTTAGAATCGTGGGCTAGAGATAACCC
TTAAGCTTCAGTTCTTTGAACCTGCATCCATATCTGTGAAATGCCTTGCAGAGTTGGGGGAAGGCTAGAGATAACCC

96

(8) 129147354 | 130282672 (8)

GCAACAGAGACCTATGTATGACCAACAAAGCCAAAGAGTAATTACTATCTGATCCTTTACAGAAAGAGTTTGCTGAC
GCAACAGAGACCTATGTATGACCAACAAAGCCAAAGAGTATGCTGACCTCTTATTAATTAAATATTTCTTTTATCGC
GAAAGTAGGAGGCAGGATGAGATTATCCCAAAGTGTCAGCATCTGACCTCTTATTAATTAAATATTTCTTTTATCGC

97

130313622 | 131901429 (8)

CCAAGCCAGCACCACAAGAACTGCTGAAAGGAGCTCTAAATCTTGAAATAAATTCTTGAAACACACCAAAATAGAAC
CCAAGCCAGCACCACAAGAACTGCTGAAAGGAGCTCTAAATCAGTTTAGAAAATGTAGATCCATTATTTGTTTACTG
ATGATGAGATGTTTCATTACAAAGTGGTTCTGTGGACCAATCAGTTTAGAAAATGTAGATCCATTATTTGTTTACTG

98

130361130 I 128711699 (8)

ACTGCATATTTGTTCAGTCTGATAATTTTTGTCTTTTATCAGTTTTCATCATTTACATTTAGTGTGATTAT

ACTGCATATTTGTTCAGTCTGCCTCCCAAAGTGCAAATGTAAAAGACAAAATAATAATGAAACCTCAAATA

AAGCCGTTTTTCTCCCCTCTTGCAGAAAATGACTAAATGTAAAAGACAAAATAATAATGAAACCTCAAATA

99

(8) 131110042 | 128401250 (8)

CTCTCTCCCAGGAACTGACATTCTCTTCTCACTGAAGCTCCCTTATGGGAAATCGATATATTCTTAAA

CTCTCTCCCAGGAACTGACATTCTCTTCTCACTGTGTTAGATGTAAGACTCCACCTCTTGAAGTGAGC

TCTGCCACATTCTATTAAACAATCACTACTGTCAGCCTAGATGTAAGACTCCACCTCTTGAAGTGAGC

100

(8) 131312646 | 131654660 (8)

CACTACTGCTGTTTTTTAATTTTTTCAAGGTAAAAACTCTCTGCCTTGTGTAGCTGGTATAACCATATTGTGGAA

CACTACTGCTGTTTTTTAATATATTGGTGATATATTAAATATATATATCACTGGCACGTGGTCAGAACTCAATAT

CCTCTGACCACACCCTCCTCCCGTGCTCATCAGCCGGGCAGACTCAGCATCTGGCACGTGGTCAGAACTCAATAT

101

(8) 131633189 | 128724024 (8)

AAGGACTTTGTCTTGTGGCTAGCATGCCAGCTCACCCACAGTAGAAAAGAGCACCAGGTATATTTCCAAGGTTCT

AAGGACTTTGTCTTGTGGCTAGCATGCCAGCTCACCAACTAGTACTCCTCAAAACTGTCAAAGAAAGCAAAGAAA

CCAAACACAAATTGAAGGATACTTTACAAAATACTTAACTAGTACTCCTCAAAACTGTCAAAGAAAGCAAAGAAA

102

(8) 131684708 | 132072532 (8)

AAATCCAGTTCCTCGGAAGAAATATCTACTAAGGACTTATATACAGAACCGAAGCCTTGGAGGGCCACAAAATGG

AAATCCAGTTCCTCGGAAGAAATATCTACTAAGGACTAGCAGGCTATTATAATAACCTTAATTTTACTCCAGCCT

AAATCCAGTTCCTCGGAAGAAATATCTACTAAGGACTAGCAGGCTATTATAATAACCTTAATTTTACTCCAGCCT

103

(8) 131698823 |131074186 (8)

GAAGTGCTCTTTGCAGCTGAGAGCCTCAGAATCTTTCTTATCACCATTATCAAGGTCAGCACTTGTCAC

GAAGTGCTCTTTGCAGCTGAGAGCCTCAGAATCTTTCTGTGTGAATTTGGGCAAATTACACAACCTCTC

GGACATGTCTCCAATTTTAGCTCCACCACTTACTAGCTGTGTGAATTTGGGCAAATTACACAACCTCTC

104

(8) 132071274 | 130684473 (8)

TAGTTTGGACAGAAAGAAGATAAAAGAACTATCACTAGAAGAGCATAGCAACCACAGATAGAACAGAAGGATCCA

TAGTTTGGACAGAAAGAAGATAAAAAAACTATCACTAGTTATAGTTTGCCCTCCAGATCCATTTTCTACCCTTCT

TGAATTCTGAAGCACAACTTGGATCTTGTGTTGGATAATCATAGTTTGCCCTCCAGATCCATTTTCTACCCTTCT

105

(18) 73918393 |131412731 (8)

AAGAAAGAAACGTGAATGCAGCTCATAATGATGATGGATTACAACATGCGGAATCATGGCCAGCGAGTTGGCCAAAT

AAGAAAGAAACGTGAATGCAGCTCATAATGATGATGGATTCCACGTTACCTCTTGGGAGAGGTAGAGATATGATTTA

TGAGCCACCGTGCCCACCTGGCCTTTTTTTTTTTCCTATGCCACGTTACCTCTTGGGAGAGGTAGAGATATGATTTA

Figure S2

Characterisation of the sequences at junctions

A. Microhomologies at junctions

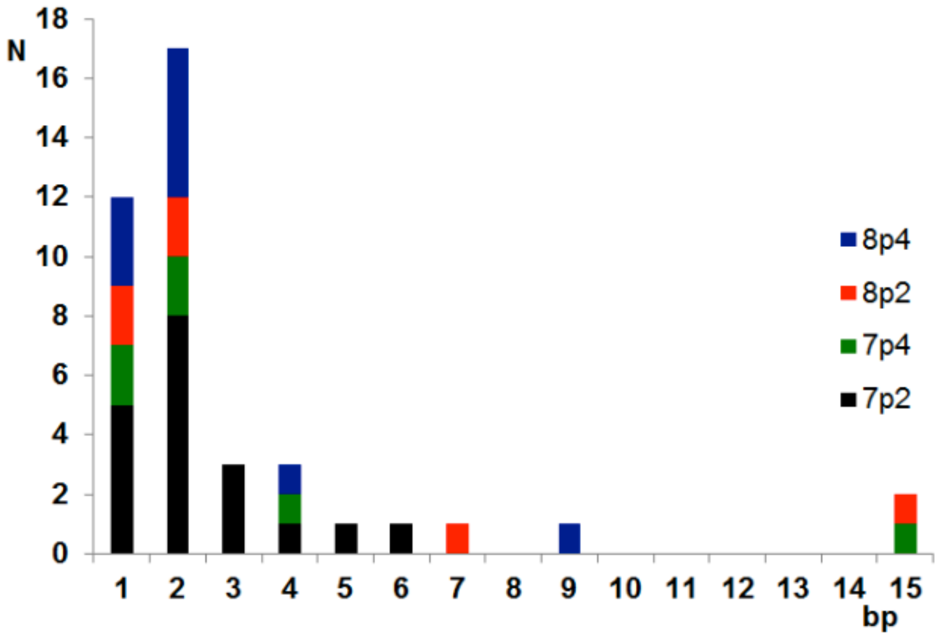

B . Insertions at junctions

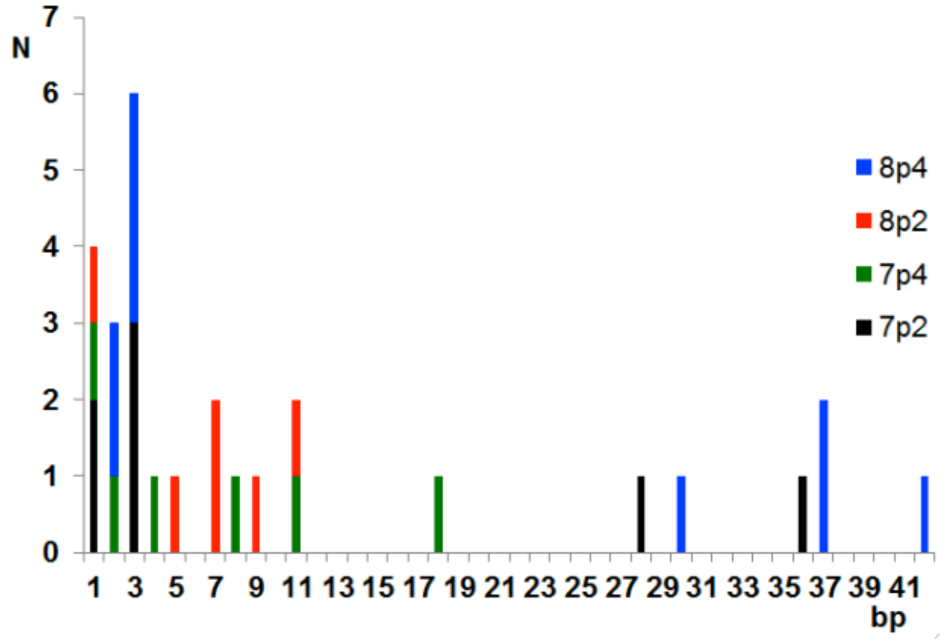

C. Origin of the sequences inserted at junctions

Junction	Insertion sequence	Localisation in the genome	
		Chromosom e	Position
7	CTAACATATGCATAACAGATGAGTGGGAAAGAAACC	12	126,321,759
27	GTATTCATATGCATAATGGCATATGAA	-	-
87	TTGGAAGCTGTATATACCTGTAATGTATGGTATTTGT	13	61,169,806
92	TCTGCCAGACACTTTGACTTTGAGAGTTAATATGTCATGTAG	9	20,348,041
95	TGGCACCCAACTTTTCACTGTACTTCTTAGAATCGTG	-	-
100	ATATTGGTGATATATTAAATATATATATCA	8	84,950,150

Supplementary figure S3

Segments of contig 18 included in non-amplified regions.

A

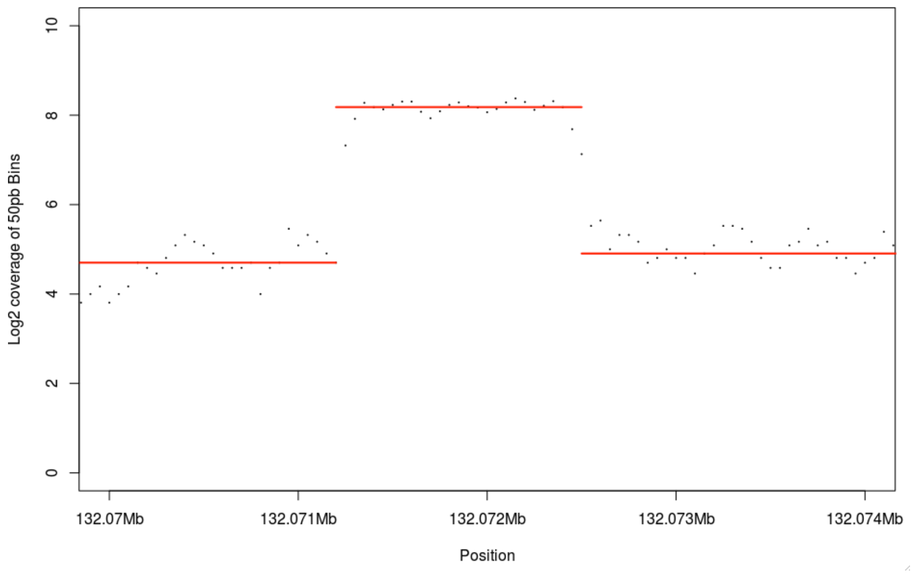

B

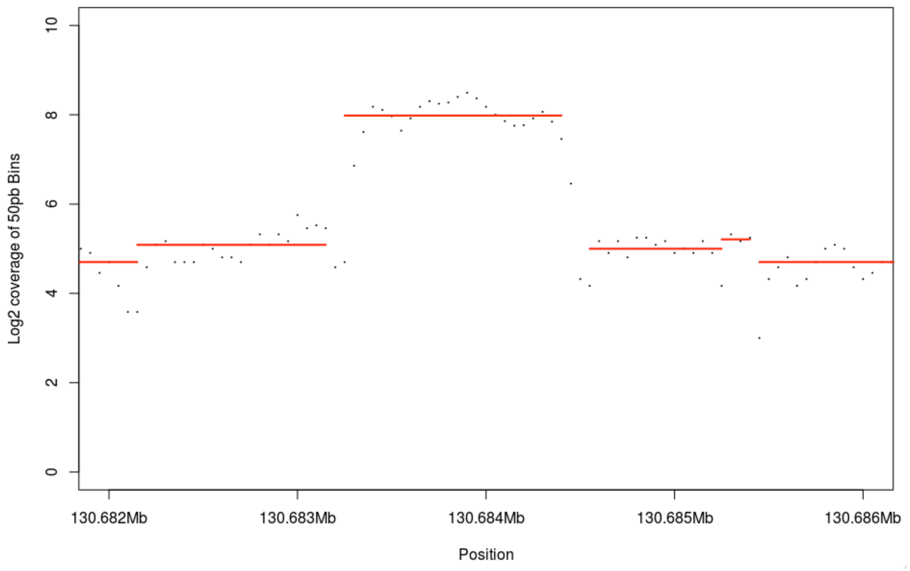

Supp Data 1.

The extenT of the *EGFR* and *MYC* gene region amplifications in ODA14 was established using the Affymetrix Genome-Wide Human SNP Array 6.0. Samples were processed at the Genomic Platform of the Institut Curie following the instructions provided by the manufacturer. Data were normalised, analysed and visualised using Partek Genomic Suite version 6.6 (Partek, St Louis, MO). Parameters used for visualisation were: minimum genomic markers: 10, signal to noise: 0.3, p-value threshold: 0.001. SNPs with smoothing values lower and greater than 2 ± 0.28 were considered as gain and loss, respectively. Microarray data were recorded in the ArrayExpress database (accession: ODA14p2, E-MEXP-3279; ODA14p4, E-MEXP-3280).

In Figure 1, amplified regions of chromosomes 7 and 8 in ODA14p2 (A and C) and ODAp4 (B and D) are presented. The amplified regions are in dmins in ODA14p2 and in an hsr in ODA14p4. The DNA copy number (CN) is reported as a function of the position on the chromosome (in Mb). The regions with 2 copies are in yellow and the regions with gains of copies in red. Three copies of chromosome 7 and 2 copies of chromosome 8 were present in the cells (see Figure 1 main text). In ODA14p4, a gain of less than one copy was observed in the region 131.33 - 131.84 Mb of chromosome 8, indicating heterogeneity in the amplicons of the hsr.

Variations in the DNA copy number were observed within the *EGFR* and *MYC* loci. However, in regions where the copy number differences were low, the position and the extent of some of these variations were dependent on the parameters used to visualise the data. Thus, a part of these variations may be poorly defined (not shown).

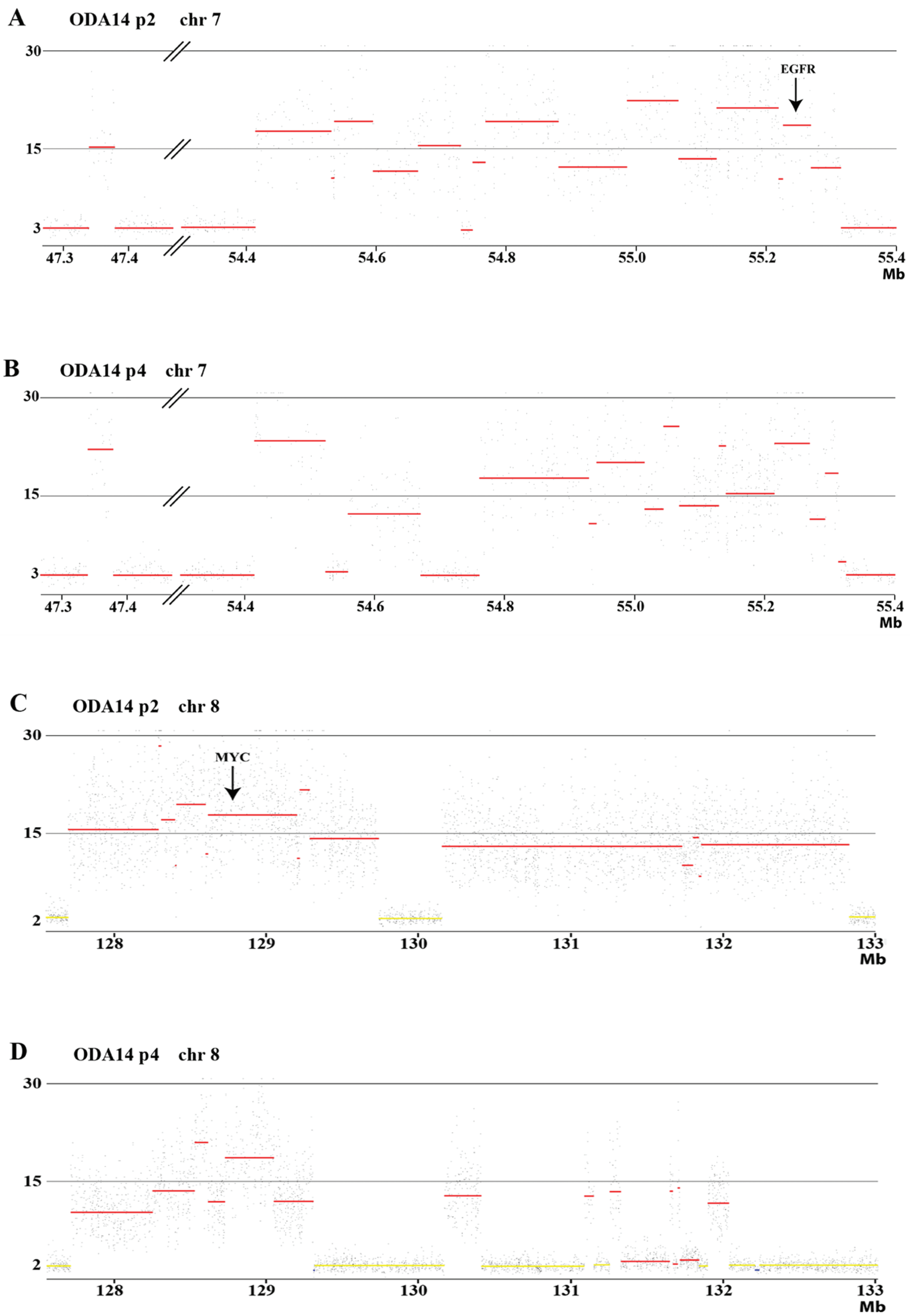
A ODA14 p2 chr 7
30
15
3
EGFR
47.3 47.4 54.4 54.6 54.8 55.0 55.2 55.4
Mb
B ODA14 p4 chr 7
30
15
3
47.3 47.4 54.4 54.6 54.8 55.0 55.2 55.4
Mb
C ODA14 p2 chr 8
30
15
2
MYC
128 129 130 131 132 133
Mb
D ODA14 p4 chr 8
30
15
2
128 129 130 131 132 133
Mb

Figure 1

Supplementary data 2

Variants

The junctions 50, 60 and 57 in ODA14p2 and 84 in ODA14p4 resulted from the deletion of segments of 8,391, 2,942, 1,999 and 2306 bp, respectively, and the sequences involved were mapped at the ends of a known structural variant (Figures 1 and 2). As normal tissue of the patient was unavailable, it was not possible to establish if junctions 50, 57 and 60 correspond to constitutional variants or if deletions occurred during dmins formation. Incontrast, since junction 84 was not present in ODA14p2, the deletion of the variant occurred during hsr formation. The presence of microhomologies in junctions 50,57 and 84 and of a blunt end in junction 60 iscompatible with the known characteristics of the non-recurrent copy number variants (1).

1. Arlt, M.F., Wilson, T.E. and Glover, T.W. (2012) Replication stress and mechanisms of CNV formation. *Curr Opin Genet Dev*, **22**, 204-210.

Figure 1

Coverage at junction 50, 57, 60 and 84

Junction 50

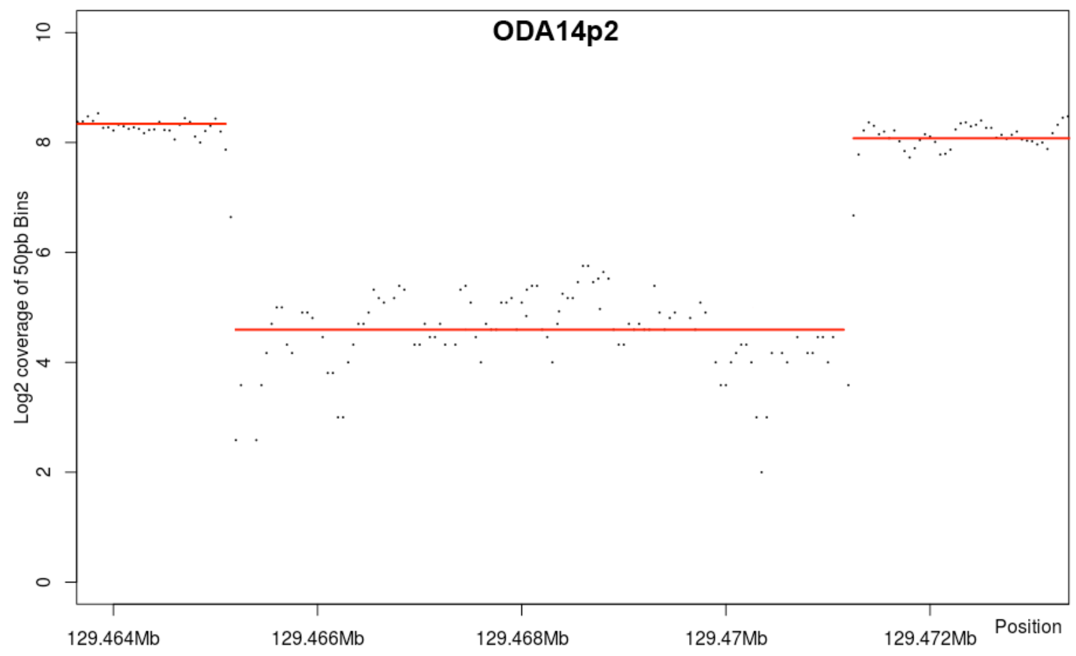

Junction 57

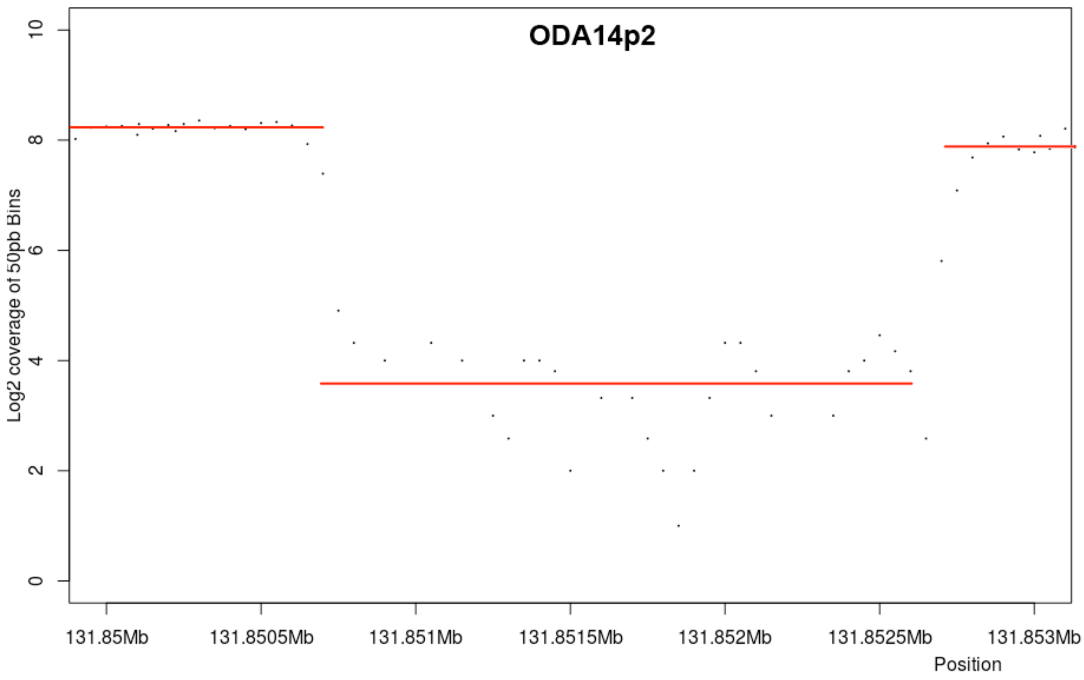

Junction 60

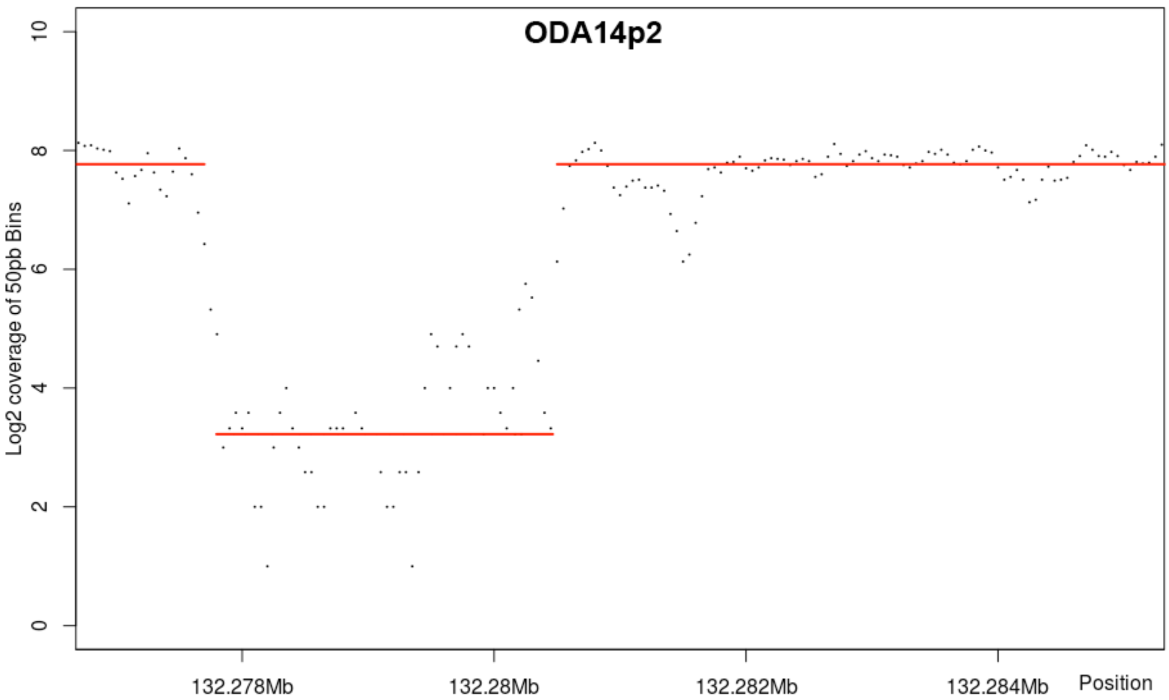

Junction 84

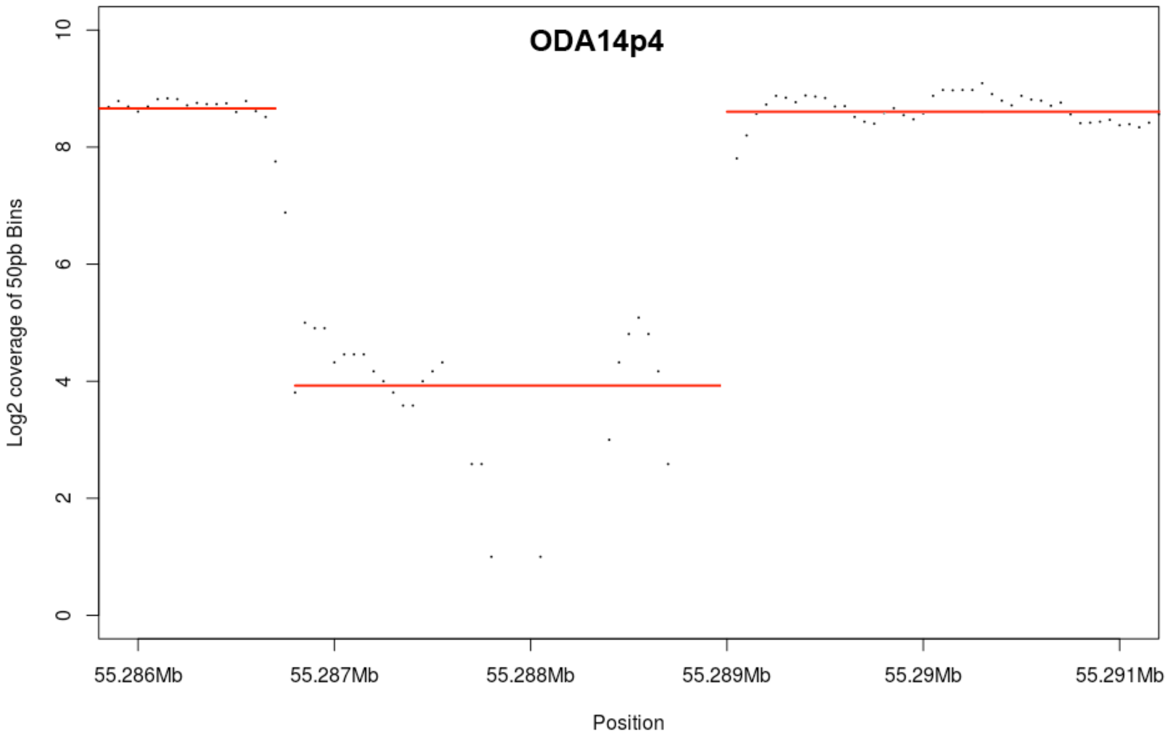

Figure 2

Localization of the junctions 50, 57, 60 and 84 in structural variants. After the human genome sequence (released February 2009) available at the UCSC Genome Bioinformatics site (http://genome.ucsc.edu/).

Junction 50 : 129,465,157 – 129,471,271

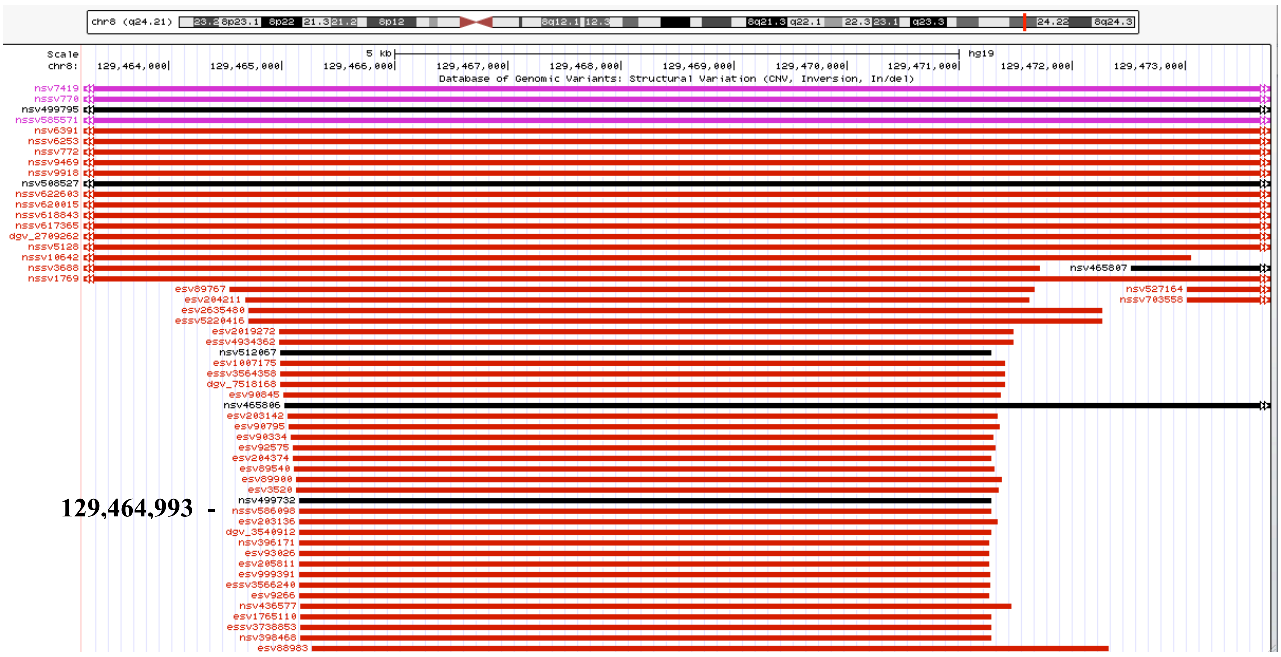

Junction 57 : 131,850,749 – 131,852,739

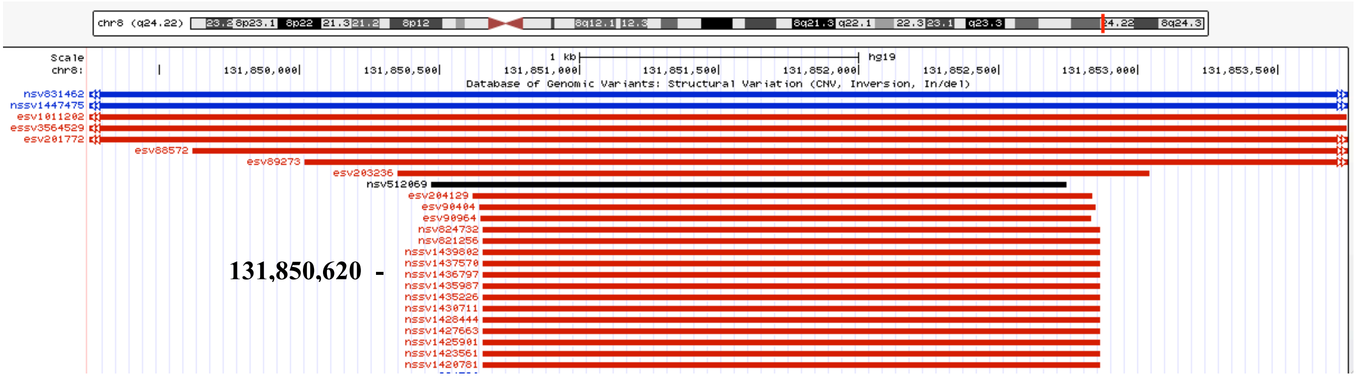

Junction 60 : 132,277,696 – 132,280,638

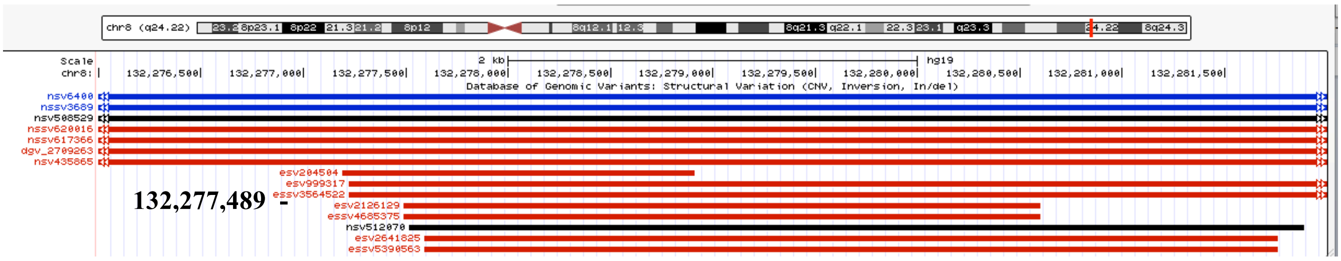

Junction 84 : 55,286,758 – 55,289,062

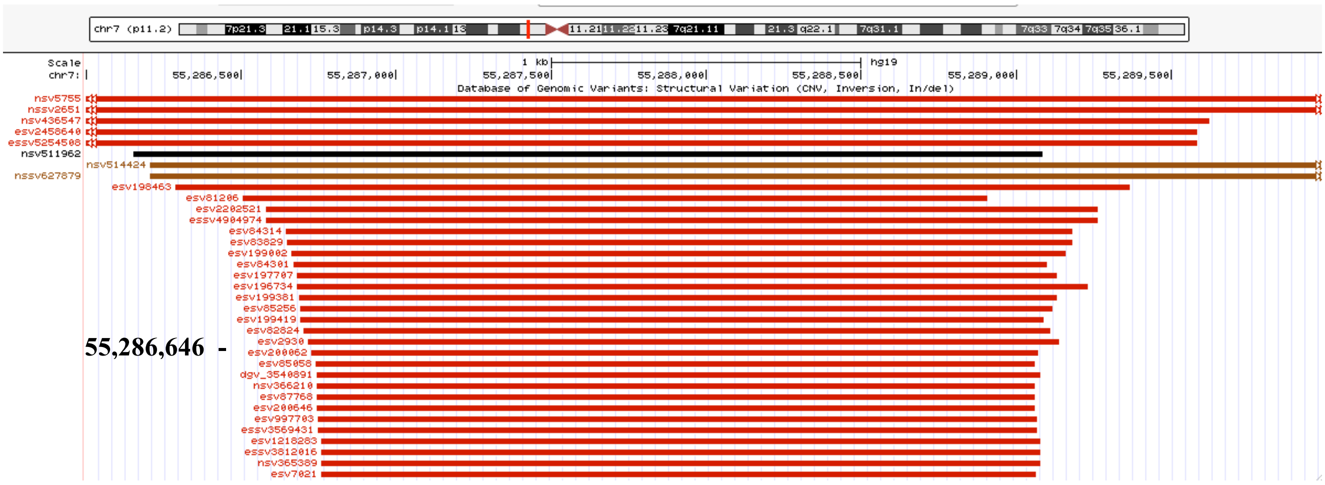

Supplementary Material and Methods

Cell cultures and fluorescent in situ hybridization (FISH).

Cell preparations were obtained after short-term culture (1-2 days) of tumour fragments. Metaphase spreads were hybridised with BAC and fosmid (Chori-BACPAC Resources) Bacterial strains containing BAC were spread on LB agar plates containing 12.5 μg mL^{-1} chloramphenicol and grown overnight at 37 °C. BAC DNA was extracted according to the manufacturer's instructions (NucleoBond Xtra Midi Plus; Macherey-Nagel). Probes were labelled with biotin (BIOT) or digoxigenin (DIG) using the BioPrime DNA labelling system (Invitrogen) and purified on Illustra ProbeQuant G-50 Micro Columns (GE Healthcare). FISH on metaphases was performed using 100 ng of BAC probes. Hybridisations were performed in 10% dextran sulphate, 50% formamide, 2SSC, 1% Tween. Immunodetection was performed by successive incubations in the following reagents: for DIG 1) FITC-conjugated mouse anti-DIG, 2) Alexa-488-conjugated goat anti-mouse; for BIOT 1) Texas-red-conjugated avidin, 2) biotin-conjugated goat anti-avidin, 3) Texas red-conjugated avidine. Slides were washed three times with PBS solution after each layer. Chromosomes were counterstained with 4′,6-diamidino-2-phenylindole (DAPI) (Vectashield mounting medium for fluorescence with DAPI; Vector Laboratories) and metaphases were observed by fluorescence microscopy. Chromosome-specific painting probes were hybridised using the protocol provided by the manufacturer (Kreatech Diagnostics).

Supplementary Table S1

Junctions found in ODA14p2. **A.** Junctions between sequences from the amplified regions of chromosomes 7 and 8. **B.** Junctions between sequences from the amplified region of chromosome 8 and other genome regions. For each arm of a junction (arm a and arm b), the strand orientation and the position, in the reference genome, of the base linked to the other arm are indicated. The exact position of the junction was established after sequencing of the PCR fragment overlapping the junction position determined by whole genome sequencing. Junctions 64 and 65 were not precisely localised by sequencing the PCR product and the indicated positions are those of the whole genome sequencing. Junctions in red remained in ODA14p4. Junctions in blue corresponded to variants. The distance in bp in the reference genome, between the arms of each junction is indicated; na: not available, fusion between chromsome 7 and 8 sequences. Microhomology: number of bp present in the normal counterparts of the fusion and maintained as a single copy the junction. Insertion: number of bases inserted between the two arms of the junction. Mapping number: number of reads of the sequence in the whole genome sequencing data.

Supplementary Table S2

Junctions found in ODA14p4. **A.** Junctions between sequences from the amplified regions of chromosomes 7 and 8. **B.** Junctions between sequences from the amplified region of chromosome 8 and from other genome regions. For each arm of a junction, the strand orientation and the position, in the reference genome, of the base linked to the other arm are indicated. The exact position of the junction was established after sequencing of the PCR fragment overlapping the junction position

determined by whole genome sequencing. Junctions in red were present in ODA14p2. The junction in blue corresponded to a variant. The distance in bp in the reference genome, between the arms of each junction, is indicated: na: not available, fusion between chromosome 7 and 8 sequences. Microhomology: number of bp present in the normal counterparts of the fusion and maintained as a single copy the junction. Insertion: number of bases inserted between the two arms of the junction. Mapping number: number of reads of the sequence in the whole genome sequencing data.

Supplementary Table S3.

Repetitive sequences involved in the junctions for sequences from chromosomes 7 and 8 **(A)** and in ODA14p2 and ODA14p4 (**B**). **C**: all junctions. No: non-repeated sequence, LINE: long interspersed nuclear elements, SINE: short interspersed nuclear elements, LTR: long terminal repeats, DNA: DNA repeat elements, Low complexity: low complexity DNA sequences. From the human genome sequence at the UCSC Genome Bioinformatics site.

Supplementary Table S4

Clusters of breakpoints in chromosome 8 present in ODA14p2 and ODA14p4. Each breakpoint is identified by the arm of the junction (Supplementary Tables S1 and S2). The distance in the normal genome between breakpoints is given in brackets. The total length of the cluster and the position of the first breakpoint are indicated.

Supplementary Table S5

Contigs observed in ODA14p2 and ODA14p4. Junctions are described in supplementary Tables S1 and S2. Chromosomes: chromosome of origin of the fragments. Length: total length in bp of the contig

Supplementary Figure S1

Sequences of the junctions in ODA14p2 and ODA14p4. The chromosome of origin of each arm and the positions of the linked bases in the reference genome are indicated. The inserted sequences are shaded grey. The microhomology sequences are in red. 103 junctions were mapped at nucleotide resolution, the sequencing of 2 junctions (64 and 65) was not possible.

Supplementary Figure S2

A. Length (N) in bp of the microhomologies at the junctions of the amplified segment of chromosomes 7 and 8 in ODA14p2 and ODA14p4. Only junctions between chromosome 7 or chromosome 8 sequences were considered. Microhomologies were found in 43% of the junctions.

B. Length (N) in bp of the inserted sequences at the junctions of the amplified segment of chromosomes 7 and 8 in ODA14p2 and ODA14p4. Only junctions between chromosome 7 or chromosome 8 sequences were considered.

C. Sequences of the inserts longer than 20 bp, the sequences in red had another localisation in the normal genome.

Supplementary Figure S3

Segments of contig 18 included in non-amplified regions. Genome sequencing coverage (50 bp bins) in the regions of chromosomes 8 in ODAp4: A 132,070,000 - 132074000 bp; B: 130,682,000 - 130,686,000 bp. The coverage of non-amplified regions is about 4. The amplified regions correspond to the segments inserted in contig 18.

Supplementary Data 1.

SNP data

Supplementary Data 2.

Characterisation of the variants

Supplementary Material and Methods.
